# Course of mental distress among psychotherapists throughout two years of the COVID- 19 pandemic: individual and inter-relational resources make a difference—cross-sectional and longitudinal results of the VOICE study

**DOI:** 10.1186/s12888-025-06867-4

**Published:** 2025-05-06

**Authors:** Sabine Mogwitz, Gloria-Beatrice Wintermann, Christian Albus, Andreas M. Baranowski, Petra Beschoner, Yesim Erim, Franziska Geiser, Lucia Jerg-Bretzke, Eva Morawa, Susann Steudte-Schmiedgen, Kerstin Weidner

**Affiliations:** 1 Department of Psychotherapy and Psychosomatic Medicine, Faculty of Medicine, Technische Universität Dresden, Fetscherstr. 74, Dresden, 01307 Germany; 2 Department of Psychosomatics and Psychotherapy, Medical Faculty and University Hospital, University of Cologne, Cologne, Germany; 3 Department of Psychosomatic Medicine and Psychotherapy, University Hospital of Bonn, University Bonn, Bonn, Germany; 4 Department of Psychosomatic Medicine and Psychotherapy, Ulm University Medical Center, Ulm, Germany; 5 Department of Psychosomatic Medicine and Psychotherapy, University Hospital of Erlangen, Friedrich-Alexander University Erlangen-Nürnberg (FAU), Erlangen, Germany

**Keywords:** COVID- 19, Psychologists, Psychotherapists, Therapists, Mental distress, Mental health, Work-related risks, Resources, Depression, PHQ- 2, Health care workers, HCW

## Abstract

**Background:**

The COVID-19 pandemic has posed challenges to healthcare systems worldwide. For healthcare workers (HCW), an increased prevalence of mental distress and the impact of various resources have been identified. Psychotherapists specialise in helping people cope with stressful life events. At the same time, they are susceptible to mental distress, resulting from their work. Data on symptoms of depression and the role of resources during the COVID-19 pandemic are scarce for psychotherapists. Therefore, the present study aimed to evaluate the course of self-reported depression of psychotherapists throughout the COVID-19 pandemic. Additionally, the impact of resources on depression was evaluated.

**Methods:**

We investigated symptoms of depression using the Patient Health Questionnaire-2 (PHQ-2) at four time points (T1-T4) during the COVID-19 pandemic in Germany. The PHQ-2 scores and resources such as sense of coherence (SOC), general optimism and social support (ESSI-D) of the psychotherapists (*N* = 1733) were compared with those of a comparison sample of HCW (*N* = 8470). The impact of resources on PHQ-2 scores was examined using cross-sectional linear modelling and longitudinal linear mixed modelling with interactions and lagged predictors.

**Results:**

At T1-T4, psychotherapists showed lower mean PHQ-2 scores than the comparison sample (*p* < 0.001). Among psychotherapists, the PHQ-2 scores increased (T1-T2, and T1-T4, *p* < 0.050). Cross-sectionally, higher SOC was associated with lower PHQ-2 scores (*p* < 0.001), with the protective influence weakening over time (*p* = 0.033). Longitudinal analyses confirmed a protective effect of sense of coherence (stable over time) and general optimism (declining over time) on PHQ-2 scores. An exploratory lagged-predictor analysis suggested that higher social support was associated with lower PHQ-2 scores, whereas higher general optimism was linked to increasing PHQ-2 scores.

**Conclusions:**

This study revealed lower levels of depression among psychotherapists compared with the comparison sample throughout the pandemic. Concurrently, the resource levels were mostly comparable and stable, with a protective impact of the sense of coherence (stable) and optimism (decreasing) and an association of high social support with low depression throughout the pandemic. Strengthening the sense of coherence and social support should be the focus of professional and policy attention to improve the ability of psychotherapists to cope with future crises.

**Supplementary Information:**

The online version contains supplementary material available at 10.1186/s12888-025-06867-4.

## Background

For nearly three years, the COrona VIrus Disease- 2019 (COVID- 19) pandemic has profoundly impacted people’s lives worldwide. The resulting mental distress has varied across different population groups. In particular, healthcare workers (HCW) have been affected [1, 2]. In addition to the unpredictable impact of the virus on health in general, HCW were immediately strained by work-related stressors such as the disruption of their regular work-routine, increased workload, insufficient personal protective equipment or other medical supplies, as well as the fear of infection and losing patients or colleagues [3–7]. Consequently, a comprehensive literature review identified working in the healthcare sector as a risk factor for increased mental burden during the COVID- 19 pandemic [8]. At the same time, recent systematic reviews on the impact of the COVID- 19 pandemic on HCW revealed higher levels of mental health problems, compared to the general population (systematic review: [9]; meta-review: [10]). The mental health of HCW during the COVID- 19 pandemic was even worse than that reported in data gathered from other pandemics or disastrous events [11]. Furthermore, elevated levels of mental distress compared to pre-pandemic scores have been identified [12], for a systematic review: [9]. More specifically, an investigation by our working group of over 3600 clinical HCW from various professions in Germany at the beginning of the pandemic, found prevalence rates of clinically significant depressive and anxiety symptoms of 17.4% and 17.8%, among physicians, and 21.6% and 19%, among nurses [13]. Notably, anxiety or depression was significantly associated with increased adverse safety outcomes in healthcare settings [14], accompanied by limitations in the quality of care [15, 16]. Individual work- and COVID- 19 related variables, such as contact with infected patients or contaminated material or being in the front line of the pandemic, have been identified specifically as risk factors for adverse mental health outcomes in HCW [17]. However, the degree of vulnerability varied among HCW, with different subgroups experiencing distinct levels of distress [13, 18].

In terms of profession, most healthcare research has focused on physicians and nurses, as well as on risk and protective factors for mental health. There is little research on other HCW professions during the COVID- 19 pandemic [19]. A subgroup whose mental health has received little research attention are psychotherapists. Psychotherapists are among those most involved in reducing the psychosocial burden of others, including patients, family members of patients and their own colleagues, not only throughout the COVID- 19 pandemic. However, the question of how the „healer heals “ him-/herself and continues to maintain good mental and physical health during the COVID- 19 pandemic has not yet received much attention [20, 21]. Being a psychotherapist is already known to be stressful and challenging per se, without being in a pandemic situation [22]. In particular, owing to the nature of their work, psychotherapists face an increased risk for emotional exhaustion and fatigue [20, 23]. Their daily work exposes them to emotionally intense experiences, including the cumulative effects of witnessing suffering, trauma, and loss [24]. In addition, administrative stressors associated with working in health services, including a lack of funding and resources, resulting in long waiting lists and clients with chronic health problems, are common [25, 26]. Even before the COVID- 19 pandemic, being young, female, or overly involved in clients'problems were identified as major risk factors for burnout of psychotherapists, followed by work-related factors, such as having less work experience [27]. Variables related to emotional interpersonal relationships at work, such as patients’ distress or distress caused by patients’ behavior, were significant stressors for clinical psychologists, as well [28]. Research dated before the COVID- 19 pandemic identified job demands, such as challenging patients or excessive workloads and insufficient resources, such as a lack of control over the work environment, professional identity or job support as predictors of burnout in psychotherapists [29, 30]. However, the COVID- 19 pandemic has introduced new challenges for psychotherapists, including social restrictions and the rapid shift from face-to-face to online therapy, often without sufficient preparation, training, or support. Recent findings suggest that lower acceptance of online therapy technology and weaker therapeutic alliances were associated with increased professional self-doubt and reduced posttraumatic growth among psychotherapists during the COVID- 19 pandemic [31]. In addition, psychotherapists had to cope with the lack of involvement of patients'families due to the rules on contact restrictions, which left psychotherapists helpless in terms of establishing social support [6, 32].

Due to the potentially severe impairment of the mental well-being of HCW and their ability to work in times of acute stress in general [33], it is crucial to identify resources that could mitigate the impact of a pandemic burden on mental health. At the beginning of the COVID- 19 pandemic, several external, individual and inter-relational resources were addressed for HCW in general, such as resilience, active and emotion-based coping strategies and social support [9]. In particular, even prior to the pandemic, psychotherapists have been suggested to be experts in the fields of functional coping, self-care and mental hygiene when confronted with acute and ongoing stressors [34]. These protective factors may explain why a recent study found that approximately 500 psychotherapists in Austria reported less mental distress during the COVID- 19 pandemic than the general population [35]. Likewise, work-related factors such as professional experience, therapeutic training, positive attitudes towards work or perceived satisfaction in helping were associated with psychotherapists’ ability to cope with stress, prior to the pandemic [20, 36]. Conversely, the lack of personal protective equipment and insufficient support from authorities and employers in cases of COVID- 19 may have increased the emotional burden of HCW in general, and psychotherapists in particular ( [37–41]; for systematic reviews: [42, 43]). In terms of individual resources, particularly for optimism, long before the COVID- 19 pandemic, multiple studies revealed positive associations with psychological and physiological well-being [44], life- [45] and work satisfaction [46] and negative associations with depression, suicide, and feelings of helplessness [45, 47, 48]. At the beginning of the pandemic, optimism was protective against burden in HCW in a large study by our work group and appeared to contribute to successful coping [49]. Moreover, optimism had a direct positive effect on work engagement in HCW during the COVID- 19 pandemic [50]. Another individual key resource for the mental well-being of psychotherapists is the sense of coherence (SOC; [36]. The sense of coherence is one of the key resilience concepts in the theory of salutogenesis, established long before the COVID- 19 pandemic [51, 52]. It can be regarded as a global orientation, reflecting the degree to which people perceive their world as comprehensible, manageable and meaningful. A systematic review of 458 studies published several years before the COVID- 19 pandemic revealed that the sense of coherence was a major predictor of the mental health in the general population, and it was also found to be negatively related to depression [52]. Among HCW in mixed professions, a higher sense of coherence was associated with fewer mental health problems before [53–55] and during the COVID- 19 pandemic [56].

In addition to these rather individual factors, inter-relational resources should also be addressed. A large systematic review revealed clear communication and high-quality social support as protective factors for mental health in the general population during the COVID- 19 pandemic ( [57–59]; for a systematic review: [43]). At the same time, social support was found to be strongly connected to lower job strain and improved health outcomes in HCW, during the pandemic [19, 60–63]. At the other end of the spectrum, lack of social support [64] or perceived loneliness were identified as significant risk factors for mortality years before the pandemic, similarl to smoking, obesity and physical inactivity [65]. An analysis of a large subsample of HCW at the beginning of the COVID- 19 pandemic from the current survey showed that a lack of social support was more strongly associated with depression and anxiety than were demographic or work-related risk factors [49]. More specifically, trust in colleagues, informal exchange and clear communication had a positive impact on the working atmosphere and were identified as important stress-reducing resources among HCW, during the COVID- 19 pandemic [13, 49, 66, 67]. Long before the COVID- 19 pandemic, the generally high impact of social support on the mental health of psychotherapists has been well studied, indicating that a supportive environment at work was among the most influential resources [36, 68]. However, there is a lack of knowledge about the impact of social support on the mental health of psychotherapists during the COVID- 19 pandemic.

To summarize, the pandemic has immediately affected the mental health of HCWs, including psychotherapists, due to both personal stressors and work-related challenges. Researchers have emphasized the need to investigate the mental health outcomes, resources, and adaptability of psychotherapists during the COVID- 19 pandemic [31, 69]. To date, research on salutogenic factors involved in maintaining the mental health of psychotherapists during the COVID- 19 pandemic is scarce. On the basis of the literature presented, we hypothesise that psychotherapists have different initial levels of mental distress of psychotherapists compared with HCW from other professions. We expect an increase in depression levels among psychotherapists over the course of the pandemic. Furthermore, we expect psychotherapists to report higher perceived resources than other HCW. We hypothesise that individual resources, such as optimism or sense of coherence, and inter-relational resources, such as social support or trust in others, have a mitigating effect on mental distress, both at the beginning and throughout the two years of the pandemic.

## Methods

### Participants and Procedures

The results of the present study are part of the ongoing prospective study “VOICE”, conducted within the framework of the egePan Unimed project ‘Development, testing and implementation of regionally adaptive care structures and processes for evidence-led pandemic management’, coordinated by university medicine [13]. The online survey assesses the stressors and resources of HCW at different time points throughout the COVID- 19 pandemic. The total sample of the prospective study “VOICE” (*N* = 23,256) so far included *N* = 8067 HCW who participated during the first time point from April 20 th to July 5 ^th^ 2020 (T1); *N* = 7190 HCW from November 17 ^th^ 2020 to January 7 ^th^ 2021 (T2); *N* = 3463 HCW from May 26 th to July 21 st 2021 (T3); and *N* = 4536 HCW from February 7 th to May 1 ^st^ 2022 (T4). The total sample of HCW comprised diverse professional backgrounds, including physicians, nurses, medical technical assistants, psychologists and administrative staff. The survey was distributed by mailing lists to all hospital staff, various professional organizations and professional online platforms. The general inclusion criteria were a minimum age of 18 years, employment in the health care sector, residence/workplace in Germany and sufficient German language skills.

For the present study, answers of a subsample of *N* = 1733 psychotherapists (in the profession of a psychologist: *N* = 1038 or physician: *N* = 695), assessed at four different time points (T1: *N* = 689, T2: *N* = 597, T3: *N* = 219, T4: *N* = 228) via an online self-report questionnaire, were analysed and compared with a large comparison sample (CS) of HCW of mixed professions (*N* = 8470), excluding psychotherapists (PT). The CS was randomly drawn from the total sample of HCW who participated in the study (without the sample of PT). To ensure a similar age and gender structure, the CS was matched by gender percentages and proportionately by age groups. In addition, a small longitudinal sample *N* = 252 was included to assess a longitudinal perspective. The inclusion criterion was the participation of the PT in at least two of the four time points. Few PT participated at three or four time points: *N* = 194 participants participated twice, *N* = 45 participants participated at three time points, and *N* = 13 participants participated at all four time points. For the exact details of the longitudinal sample, including an exact differentiation between the possible combinations of participations at time points, see Table 1S of the supplementary material.

### Ethics approval and consent to participate

The study was approved by the Ethics Committee of all the participating study centers, e.g., of the Medical Faculty of the Rheinische Friedrich Wilhelm University Bonn (reference number: 125_20), the Medical Faculty of the Friedrich-Alexander University Erlangen-Nürnberg (reference number: 133_20 B) and the Medical Faculty of the University of Cologne (reference number: 20 - 1199_4) and was registered on ClinicalTrials (DRKS-ID: DRKS00021268; registration date: 9 th of April 2020). All the respondents provided their online informed consent.

### Outcome measure of mental distress

For the present study, self-assessed symptoms of depression were assessed as the primary outcome at four time points (T1, T2, T3 and T4) during the COVID- 19 pandemic. Symptoms of depression were measured using the Patient Health Questionnaire (PHQ- 2; [70]). This ultrashort form of the Patient Health Questionnaire (PHQ_D; [71, 72]) measures depression levels over the preceding two weeks with two items. The participants indicated how often they experienced “little interest or pleasure in doing things” (item 1) and “feeling down, depressed or hopeless” (item 2) with Likert-type answers, ranging from 0 („not at all “) to 3 („nearly every day “). The aggregate sum score ranges from 0 to 6. The sum score of these two items (PHQ- 2 score) was defined as the primary outcome of the present work. Furthermore, a cut-off value of ≥ 3, which is based on the PHQ- 2 score, has been suggested to identify likely cases of clinically relevant depression [70]. The PHQ- 2 rate can be calculated as percentage of participants meeting the cut-off value, corresponding to the proportion of cases with likely clinical depression. The additional calculation of PHQ- 2 rates helps to compare our findings with the literature. The psychometric characteristics of the PHQ- 2 are well documented [70]. In the present sample, the validated German version of the PHQ- 2 obtained an acceptable Cronbach's α score of 0.766.

### Sociodemographic, work- and COVID- 19-related control variables

The online questionnaire assessed general sociodemographic variables at all four time points, of including age, gender, living alone and having children. In addition, work-related variables such as workplace, work experience and change of department were considered. As a COVID- 19- related variable, having direct contact with COVID- 19 (two items: infected patients or contaminated material) was of interest as a control variable. In the present sample, Cronbach’s α of the variable “having direct contact with COVID- 19” was 0.767.

### Potential resource variables

#### Individual resources

*General optimism* during the COVID- 19 pandemic was considered as a potential individual resource variable and was measured using a single item “How optimistic are you in general?” [73], with answers ranging from 1 (“not optimistic at all”) to 7 (“very optimistic”). General optimism was assessed at all four time points.

In the present study, the sense of coherence (SOC) was assessed using a German ultrashort version (SOC- 3; [74]) of the original SOC scale developed by Antonovsky [51]. The SOC- 3 is a highly economic instrument with sufficient reliability that shows strong correlations with the original SOC scale [74]. Only two of the three subscales from the original version are present in the SOC- 3, namely, comprehensibility and meaningfulness, measuring the SOC on the basis of three items. Two of them are rated on a scale ranging from 1 (“very often”) to 7 (“very seldom or never “), e.g., “Do you have very mixed-up feelings and thoughts?”. The third item is scaled from 1, “Do you feel how good it is to be alive?” to 7, “Do you ask yourself why you exist at all?” and has to be inverted. The final SOC- 3 sum score therefore ranges from 3 to 21. Higher values in the SOC- 3 indicate a stronger SOC. In the present sample, Cronbach’s α was 0.708. SOC was assessed at all four time points.

Potential work-related resource variables during the COVID- 19 pandemic- Of the up to 7 items captured within the original survey (six items at T1, seven items at T2, and five at T3 and T4), in the present study, four items, considered as potential resources, were investigated: „There is sufficient personnel protective equipment for the staff, including mouth protection “ (only assessed at T1 and T2), „There is sufficient staff.“ (T1-T4), „I can recover sufficiently during spare time.“ (T1-T4) and „I can trust in my colleagues during difficult times at work.“ (T1-T4). Despite the variable „There is sufficient personnel protective equipment for the staff, including mouth protection.“ (only assessed at T1 and T2), the other variables were assessed at all four time points. The items were rated separately on a single scale, ranging from 0"strongly disagree"to 4"strongly agree", and refer to the preceding two weeks. For further details on the assessed variables see Table 2S of the supplementary material.

Potential COVID- 19-related resource variables during the COVID- 19 pandemic- Of the 16 items captured in the original survey, two items, „I felt protected by local authorities.“ and „I felt protected by my employer.“, were assessed at all four time points. The two items, which were measured separately on a single scale from 0"strongly disagree"to 4"strongly agree", with respect to the preceding two weeks [75], were assessed at all four time points.

For both, potential work-related resource variables and potential COVID- 19-related resource variables, consent „yes “ was presumed, if participants had quoted either 3 („rather agree “) or 4 (“strongly agree “). For further details on all the assessed variables see Table 2S of the supplementary material.

*Social Support* was measured using the German version of the ENRICHD Social Support Inventory (ESSI-D; 76). The ESSI is a five-item questionnaire (e.g. “Is there someone available to give you advice about a problem?”), answered on a 5-point Likert scale (1 = never, 5 = all the time), with a score ranging from 5 to 25. A cut-off value of ≤ 18 and an answer of at least two items ≤ 3 are indicative of low social support [76]. Kendel et al. [76] reported a Cronbach’s α of 0.89 for the ESSI-D, which is in line with the Cronbach’s α score of the present sample (0.901). Social support was assessed at all four time points.

### Statistical analyses

All statistical analyses were conducted with SPSS Version 29 and the programming language R V 4.2.0 [77, 78], using the packages lme4, tidyr, dplyr, and car [79–81]*.* Descriptive statistics and chi square (χ^2^)-tests (relative frequencies for categorical variables) were calculated to describe the sociodemographic characteristics of the study sample, psychotherapists (PT) and the comparison samples (CS).

Dropout analyses were conducted comparing cross-sectional and longitudinal PT participants, including all variables of interest (sociodemographic, outcome and resource variables). Moreover, comparisons were made of sociodemographic variables within the longitudinal sample of PT at each time point in relation to the number of participations. Further details can be found in Table 3S in the supplementary material.

For cross-sectional comparisons between PT and CS (e.g., the PHQ- 2 sum scores or sum scores of the resource variables general optimism, SOC and ESSI-D), a two-sample *t*-test for continuous variables was performed. The χ^2^-test was performed for categorical variables such as the PHQ- 2 rates and categorical resource variables (e.g., consent with sufficient recovery, trust in colleagues). In the cross-sectional analyses, no participant was assessed twice: participants who participated at more than one time point were only included at their first time of participation, and subsequent data with the same code were excluded from the cross-sectional analysis to avoid bias and overestimation of effect sizes. The effect sizes (Cohen's d and Cramer´s V) are reported (0.2 ≤ d < 0.5: small, 0.5 ≤ d < 0.8: medium, d ≥ 0.8: large effect size; 0.1 ≤ V < 0.3: small, 0.3 ≤ V < 0.5: medium, V ≥ 0.5: large effect size; [82]). To examine group differences within the cross-sectional samples with respect to time points T1, T2, T3, and T4, univariate analyses of variance (ANOVA) were conducted. The effect sizes η_p_^2^ (partial eta-squared) are reported (0.01 ≤ η_p_^2^ < 0.06: small, 0.06 ≤ η_p_^2^ < 0.14: medium, η_p_^2^ ≥ 0.14: large effect). The results were followed up by *post-hoc* analyses* (*Games-Howell post hoc test, to account for unequal variances*)*. To address the problem of multiple testing, we stringently considered the Bonferroni-corrected *p*-values (p/number of tests, separately for the t-tests and χ^2^-tests, for each sample investigated).

To analyse the effects of the potential resources of PT on the PHQ- 2 at the four time points, linear modelling was used for the cross-sectional sample (analysis 1), and linear mixed modelling with interaction (analysis 2) and lagged predictors (analysis 3) was used for the smaller longitudinal sample. External resources, such as sufficient gear or sufficient staff, individual resources, such as general optimism, SOC and recovery during spare time, and inter-relational resources, such as the ESSI-D, trust in colleagues and protection by local authorities and employer, were considered as potential assets.

For the cross-sectional analysis (analysis 1), a linear model (LM) was used to analyse data collected at four distinct time points (T1, T2, T3, T4) cross-sectionally from separate samples of participants (*N* = 1733, divided up into four subsamples, one at each time point). This approach allowed us to assess the impact of the resources on PHQ- 2 scores at each time point independently. The model fit was assessed using maximum likelihood estimation (for a detailed description of the model selection process, see Table 5S of the supplementary material).

For participants who took part in at least two time points (longitudinal sample, *N* = 252), longitudinal analyses (analyses 2 and 3) were conducted using linear mixed-effects models (LMM). This approach considers repeated measures within participants over time and includes interaction terms between predictors and time points, e.g., time point * sufficient gear (analysis 2), to investigate how the effects of resources vary longitudinally. Additionally, an exploratory LMM was performed with lagged predictors, e.g., lag(sufficient gear, *N* = 1) within analysis 3, to assess the influence of these predictors on PHQ- 2 scores at subsequent time points. For a detailed description of the model selection process, see Table 6S of the supplementary material.

The influence of each variable was assessed by using estimates with confidence intervals (CI), standard errors (SE), t- and *p*-values. A negative estimate with a significant *p*-value indicates a protective effect of the respective resource/control variable on the primary outcome (PHQ- 2 score). A level of significance of *p* ≤ 0.050 (two-tailed) was determined in all analyses. The coefficients and test statistics are presented in Table 4 for the cross-sectional sample and Tables 5 and 6 for the longitudinal sample. For both samples, only sociodemographic control variables that correlated significantly with PHQ- 2 scores (*p* ≤ 0.050) at the time point of interest were included (exact values of correlation analyses; Table 7S of the supplementary material for control variables and Table 8S for the resource variables of interest). A diagnosis concerning the multicollinearity of variables was taken into consideration using variance inflation factors as indicators. Multicollinearity did not exist in any linear model**.** For a detailed description of the model selection process see Tables 5S and 6S of the supplementary material.

## Results

### Description of the total study sample and results of the dropout analyses

#### Description of the total study sample

A total of 1733 psychotherapists (*N* = 1038 in the profession of a psychologist, *N* = 695 in the profession of a physician) were assessed during four waves (T1: *N* = 689, T2: *N* = 597, T3: *N* = 219, T4: *N* = 228) and compared to a comparison sample of 8470 HCW of mixed professions, excluding psychotherapists. Comparted with the age- and gender-matched comparison sample, psychotherapists tended to live alone less often, had children in their own household more often, had more work experience, were working more often in direct patient care, worked less often in a university hospital and had less contact with COVID- 19 patients or contaminated material (*p* ≤ 0.006). For further sociodemographic, work- and COVID- 19 related details of the study sample see Table 1. For a description of the small longitudinal sample, see Table 1S of the supplementary material.

**Table 1:**
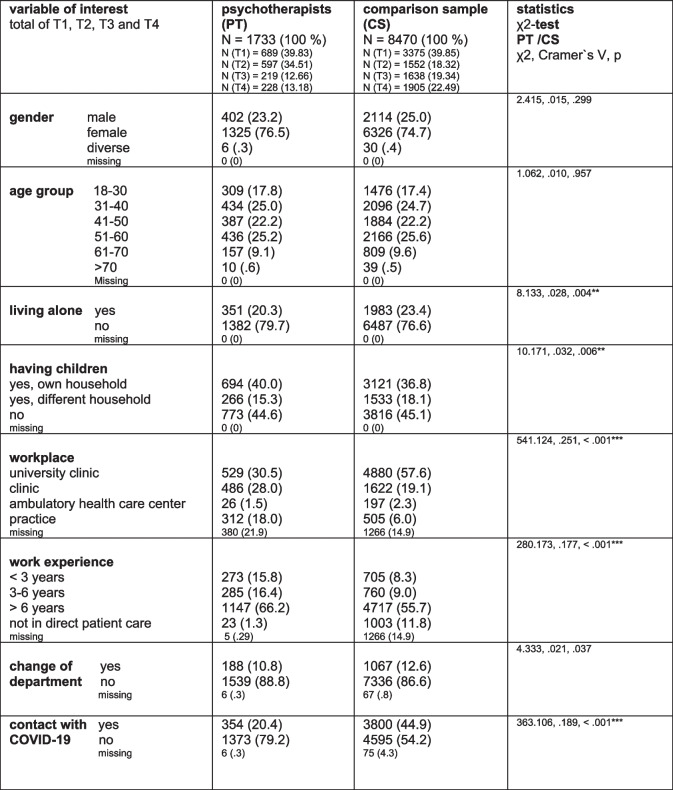
Description of the total study samples (psychotherapists and comparison sample)

significant *p*-values are marked: *: *p* ≤.050, **: *p* ≤.010, ***: *p* ≤.001*; Bonferroni correction: *p* ≤.050/8 (.006) for 8 sociodemographic variables assessed within the same analysis; T1, T2, T3, T4 = time point 1, 2, 3 and 4

#### Results of the dropout analyses

The cross-sectional and longitudinal samples of psychotherapists at T1 were mostly comparable. Furthermore, participants in the longitudinal sample did not differ on any of the sociodemographic variables of interest at any of the four time points depending on whether they had participated 2, 3 or 4 times. However, few differences with very low to low effect sizes were identified. In the cross-sectional sample at T1, more participants had children (*p* = 0.002) than in the longitudinal sample. For details, see Table 3S of the supplementary material. Longitudinal participants more often worked in university clinics than cross-sectional participants did at T1 (*p* < 0.001) and more often felt protected by local authorities (*p* = 0.002). The PHQ- 2 score and rate by tendency were lower for longitudinal participants at T1 (*p* = 0.028; 0.034; not significant after Bonferroni correction). However, none of the sociodemographic variables correlated significantly with PHQ- 2 scores in the cross-sectional or longitudinal analyses. For exact details of the longitudinal comparisons, see Table 4S, and for the correlation analyses, see Table 7S of the supplementary materials.

### Depression of psychotherapists and the comparison sample at T1, T2, T3 and T4 and the course of depression: cross-sectional samples

#### Depression of psychotherapists (PT) compared with the comparison sample (CS): cross-sectional analyses

**Fig. 1:**
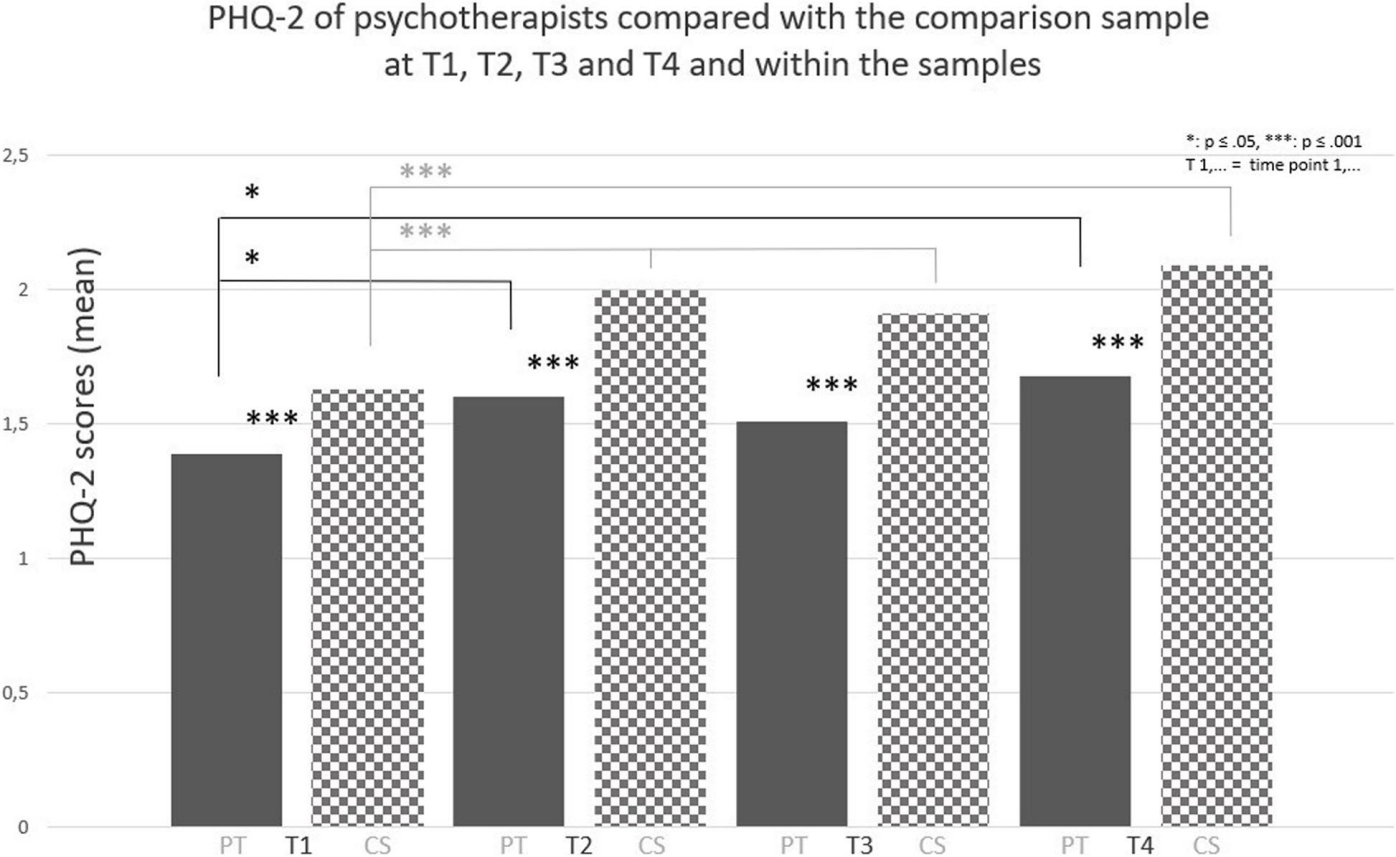
PHQ- 2 scores of psychotherapists (*N* = 1733) compared with the comparison sample (*N* = 8470) at T1, T2, T3 and T4 and within the samples

At T1, T2 and T3, PT showed less symptoms of depression (PHQ- 2 score) than the CS, as well as lower rates of clinically relevant depression with a cut-off of the PHQ- 2 sum score of ≥ 3 (*p* < 0.001). At T4, although PT also showed lower severity of depression than the CS (*p* < 0.001), the descriptively lower rates of clinically relevant depression did not reach statistical significance after Bonferroni correction. For further details, see Table 2 and Fig. 1.

**Table 2:**
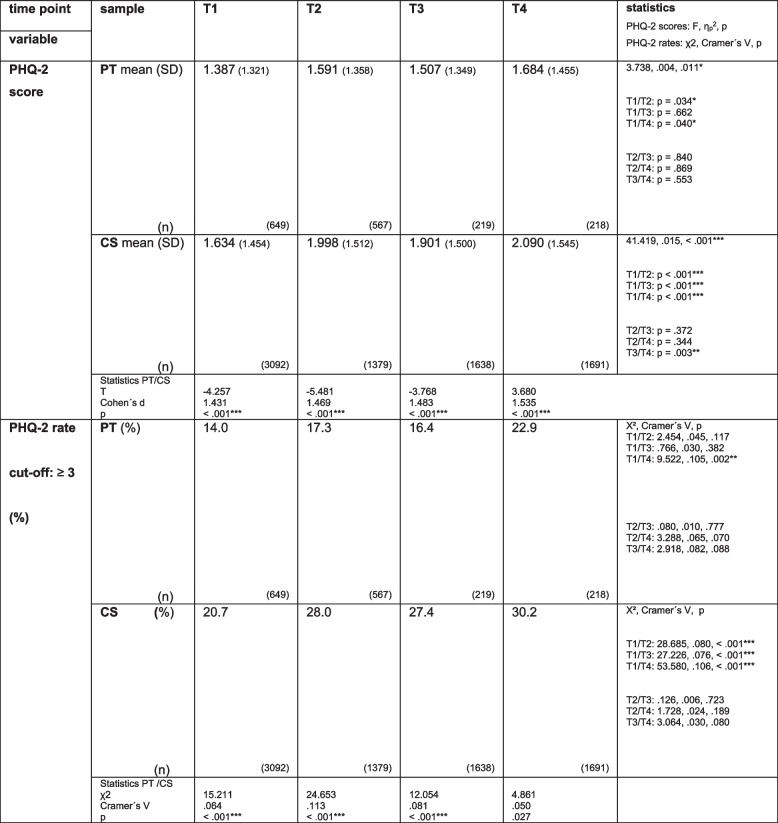
PHQ- 2 score of psychotherapists (PT) and PHQ- 2 rates (based on the PHQ- 2 cut-off score: ≥ 3) compared with the comparison sample (CS) at T1, T2, T3 and T4: cross-sectional samples

PT = psychotherapists; CS = comparison sample; significant p-values are marked: *: *p* ≤.050, **: *p* ≤.010, ***: *p* ≤.001; Bonferroni correction: *p* ≤.050/4 (.013) for 4 score variables with means: PHQ- 2 score, general optimism, SOC, ESSI-D and *p* ≤.050/7 (.007) for 7 categorial variables: PHQ- 2 rate + 6 resource variables; PHQ- 2 = separate module of the PHQ- 4 (Patient Health Questionnaire) assessing depression; cut off (PHQ- 2 score) for clinically relevant depression: ≥ 3; T1, T2, T3, T4 = time point 1, 2, 3 and 4

#### Course of depression within the cross-sectional samples of PT and the CS throughout the pandemic at T1, T2, T3 and T4

For psychotherapists, levels of depression increased from T1 to T2 (*p* = 0.034) and from T1 to T4 (*p* = 0.040). The rates of clinically relevant depression tended to increase from 14.0% at T1 to 17.3% at T2 (*p* = 0.117), stabilised at 16.4% at T3 (*p* = 0.382) and increased again to 22.9% at T4 (*p* = 0.002). According to the comparison sample, levels of depression increased from T1 to T2, from T1 to T3, and from T1 to T4 (all: *p* < 0.001). The rates of clinically relevant depression increased from 20.7% at T1 to 28.0% at T2 (*p* < 0.001), stabilised at T3 and increased again to 30.2% at T4 (*p* < 0.001). For further details see Table 2 and Fig. 1.

### Resources of psychotherapists (PT) compared with those of the comparison sample (CS) and within psychotherapists throughout the pandemic at T1, T2, T3 and T4

#### Resources of PT compared with those of the CS at T1, T2, T3 and T4

The levels of general optimism, SOC and ESSI-D were mostly comparable between PT and CS at T1, T2, T3 and T4. However, PT reported a higher ESSI-D at T1 (*p* = 0.010) and a higher SOC score at T2 (*p* = 0.009). At T1, PT more often agreed that protective gear was sufficient (*p* < 0.001) and perceived protection by local authorities than the CS (*p* < 0.001). At T2, PT more often agreed with sufficient staff and that protection by local authorities and their employer was sufficient (all *p*-values < 0.001) and, by trend, that recovery during spare time was sufficient (*p* ≤ 0.008) but not significant after Bonferroni correction, with comparable agreement with respect to trust in colleagues and sufficient protective gear. At T3 and T4, PT more often agreed that they were protected sufficiently by local authorities (*p* < 0.001). The Bonferroni correction was applied as follows: *p* ≤ 0.050/4 (0.013) for four score variables with means and t-tests: PHQ- 2 score, general optimism, SOC, ESSI-D, and *p* ≤ 0.050/7 (0.007) for seven categorical variables and χ^2^-test: PHQ- 2 rate + six categorical resource variables*.* For further details on the resource variables with means of sum scores and for variables assessed as rates of consent (in %), see Table 9S of the supplementary material.

#### Course of resources within the cohort of psychotherapists throughout the pandemic at T1, T2, T3 and T4

**Fig. 2:**
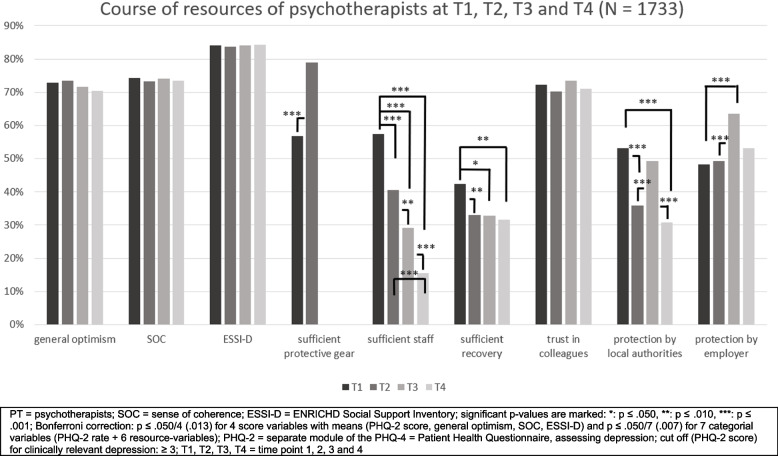
Course of resources of psychotherapists at T1, T2, T3 and T4 (*N* = 1733)

Throughout T1, T2, T3 and T4, PT reported stable levels of general optimism, SOC, ESSI-D and consent with trust in colleagues. Consent with sufficient protective gear increased from T1 to T2 (*p* < 0.001), whereas consent with sufficient staff decreased from T1 to T2, T3 and T4 (*p* < 0.001), T2 to T3 (*p* = 0.004), to T4 (*p* < 0.001), and T3 to T4 (*p* < 0.001). Consent with sufficient recovery during spare time decreased from T1 to T2 (*p* = 0.002), stabilized to T3 and decreased again to T4, with a general decrease from T1 to T4 (*p* = 0.004). Consent with perceived protection by local authorities decreased from T1 to T2 (*p* < 0.001), increased from T2 to T3, and decreased from T3 to T4, at a significantly overall decreasing level from T1 to T4 (all: *p* < 0.001). Consent with perceived protection by employers increased from T1 to T3, from T2 to T3 (both: *p* < 0.001) and stabilized from T3 to T4. For further details on the variables of resources with means of sum scores and for variables assessed as rates of consent (in %), see Table 3 and Fig. 2.

**Table 3:**
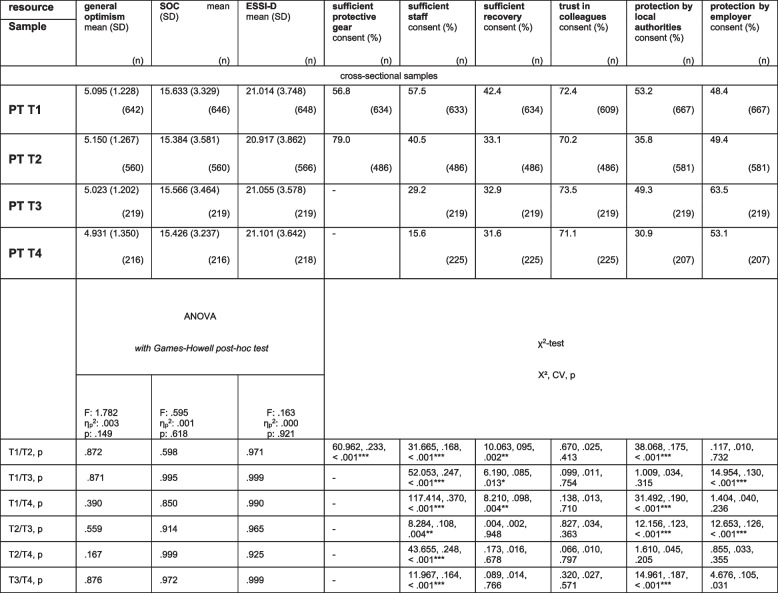
Parameters of external, individual and inter-relational resources of psychotherapists at T1, T2, T3 and T4: cross-sectional samples

SOC = sense of coherence; ESSI-D = ENRICHD Social Support Inventory; significant p-values are marked: *: = *p* ≤.050, **: = *p* ≤.010, ***: = *p* ≤.001; Bonferroni correction: *p* ≤.050/4 (.013) for four score variables with means: PHQ- 2 score, general optimism, SOC, ESSI-D and *p* ≤.050/7 (.007) for seven categorial variables (PHQ- 2 rate + 6 resource variables); PHQ- 2 = separate module of the PHQ- 4 = Patient Health Questionnaire- 4, assessing depression; cut off (PHQ- 2 score) for clinically relevant depression: ≥ 3; T1, T2, T3, T4 = time point 1, 2, 3 and 4; “-“: not assessed at this time point

### Results of the linear model of external, individual and inter-relational resources and the outcome variable severity of depressive symptoms (PHQ- 2 score) of psychotherapists at T1, T2, T3, and T4 (analysis 1): cross-sectional samples (*N* = 1733)

Model selection: The decision to use a linear model (LM) was based on initial analyses using a linear mixed model (LMM) under the same conditions, which included time as a random effect. The LMM showed negligible variance for the random effect of time point (variance = 0.000, SD = 0.000), indicating no significant impact on the dependent variable. This suggested that modelling time as a random effect did not improve variance explanation. Therefore, we opted for a linear model without random effects, focusing on fixed predictors and their interactions with time. This approach allowed us to directly examine the significance of key predictors and their interactions across all time points, aligning the model with the cross-sectional nature of the data and our research objectives. For a detailed description of model selection, see Table 5S of the supplementary material. Quality of the model: A multiple R-squared of 0.419, consequently an adjusted R-squared of 0.409, indicated an explanation of variance of approximately 40.9% of the dependent variable. We consider this to be moderate, suggesting an acceptable fit of the model to the included data.

*Significant main effects:* The intercept value was significant (*p* < 0.001), given the base value of the dependent variable. SOC had a significant negative effect (estimate = − 0.227, *p* < 0.001), suggesting an association of higher SOC with lower PHQ- 2 scores.

*Interactions of time point with predictors: *The interaction effect between time point and sufficient recovery during spare time was negative (estimate = − 0.143, *p* = 0.019), suggesting a changing influence of recovery during spare time on PHQ- 2 scores during the pandemic. The interaction of time point and trust in colleagues revealed a significant negative effect (estimate = − 0.147, *p* = 0.036), suggesting a changing influence of trust in colleagues on PHQ- 2 scores during the pandemic (with increasing time points). The interaction between time point and SOC revealed a significant positive effect (estimate = 0.046, *p* = 0.033), indicating a weakening of the protective influence of SOC on PHQ- 2 scores as the pandemic progressed. For more details see Table 4.

**Table 4:**
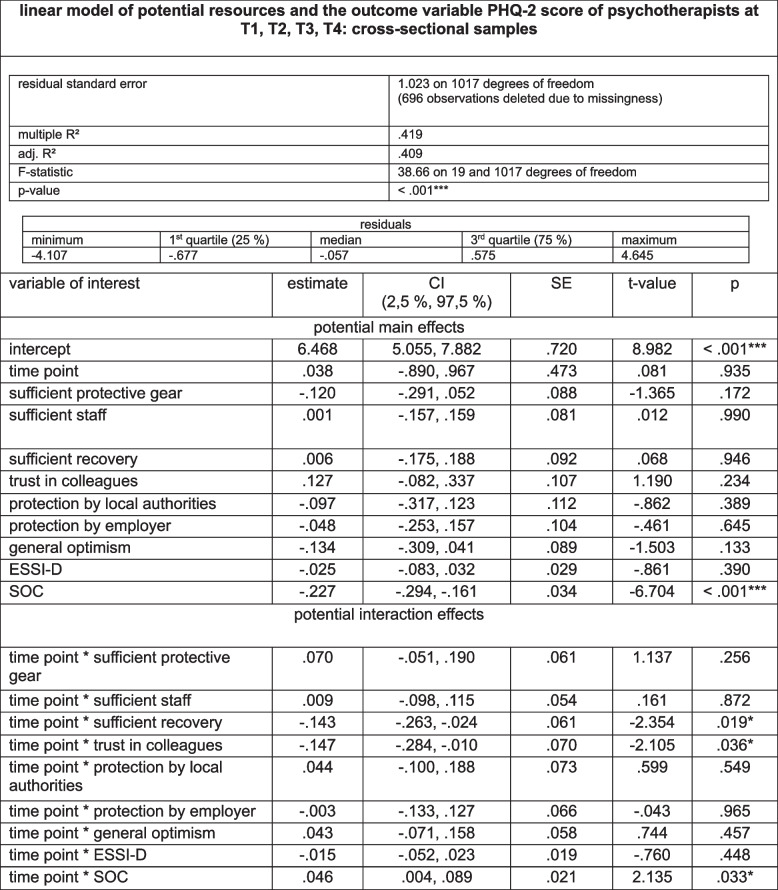
Linear model of external, individual and inter-relational resources and the outcome variable severity of depressive symptoms (PHQ- 2 score) of psychotherapists at T1, T2, T3, T4 (analysis 1): cross-sectional samples

adj. R² = adjusted R² (explained variance); SE = standard error; (manifestation coded by 0 versus. manifestation coded by 1); significant p-values are marked: *: *p* ≤.050, **: *p* ≤.010, ***: *p* ≤.001, ESSI-D = ENRICHD Social Support Inventory

*Summary:* Linear modelling of the cross-sectional sample revealed that higher SOC was associated with lower PHQ- 2 scores, with the protective effect of SOC decreasing over time. The influence of recovery during spare time and trust in colleagues on the PHQ- 2 score also diminished. The model fit was moderate, indicating a substantial proportion of explained variance among the predictors.

### Results from the linear mixed models (LMM) with interaction predictors and lagged predictors (variables of external, individual and inter-relational resources and outcome variable PHQ- 2 score for the longitudinal sample of psychotherapists at T1, T2, T3 and T4), *N* = 252

#### Results from the linear mixed model (LMM) with interaction predictors (time point * resource variable) at T1–4 (analysis 2)

Model selection: A linear mixed model (LMM) with a model fit by maximum likelihood including fixed, random and predictor interaction (time point * resource variable) effects was carried out to investigate the impact of the predictors/variables of interest on the PHQ- 2 scores at different time points. For a detailed description of model selection, see document 6S of the supplementary material. Quality of the model: For the exact specifications of the quality of the model, see Table 5.

**Table 5:**
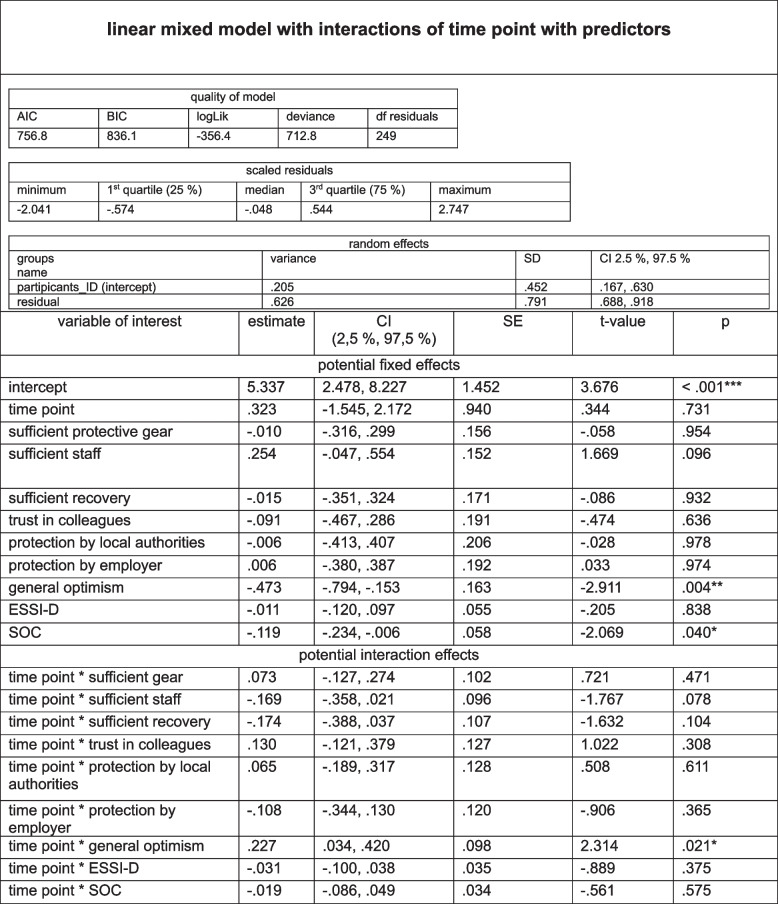
Linear mixed model of external, individual und inter-relational resources, interactions with predictors (time point * resource) and the outcome variable severity of depressive symptoms (PHQ- 2 score) of psychotherapists at T1, T2, T3 and T4: longitudinal sample

significant *p*-values are marked: *: *p* ≤.050, **: *p* ≤.010, ***: *p* ≤.001; ESSI-D = ENRICHD Social Support Inventory; CI = confidence intervals; SD = standard deviation; df = degrees of freedom; logLik = log-likelihood; AIC = Akaike Information Criterion; BIC = Bayesian Information Criterion

*Fixed effects:* The intercept was significant (estimate = 5.337, *p* < 0.001). The effect of time point was not significant (estimate = 0.323, *p* = 0.731), suggesting that the PHQ- 2 score does not significantly increase or decrease over time, independent of the other predictors. A significant negative main effect of general optimism (estimate = − 0.473, *p* = 0.004) indicated an association of higher scores of general optimism with lower PHQ- 2 scores, suggesting a protective effect of optimism against symptoms of depression. A significant negative main effect of SOC (estimate = − 0.119, *p* = 0.040) indicated an association of a higher SOC with lower PHQ- 2 scores.

*Interactions of time point with predictors:* A significant positive interaction effect time point * general optimism (estimate = 0.227, *p* = 0.021) suggests that the protective effect of general optimism decreases over time (throughout the pandemic).

*Random effects:* Participant_ID (intercept): The variance of random effects (estimate = 0.205, SD = 0.452) indicated that there was variability in the PHQ- 2 scores between the participants, which was not explained by fixed effects. Residuals: The variance of the residuals (estimate = 0.626, SD = 0.791) reflects the amount of variance that remains unexplained after accounting for both fixed and random effects.

*Summary:* The results of the linear mixed model with interactions of time points with predictors suggest that general optimism and the SOC were significant protective factors for depressive symptoms. The effect of general optimism seems to decrease over time, whereas the significant effect of SOC was stable. The other predictors and the time point did not suggest significant main effects or interaction effects. For further details, see Table 5.

#### Results from the linear mixed model (LMM) with lagged predictors at T1–T4 (analysis 3)

A linear mixed model (LMM) with lagged predictors was carried out to investigate the impact of the variables of interest at an earlier time point on the PHQ- 2 scores at later time points. The lagged predictor linear mixed model examined how the values at the previous time point for our variables of interest (sufficient equipment, staff and recovery, trust in colleagues, protection by local authorities and the employer, general optimism, ESSI-D, and SOC) influenced subsequent PHQ- 2 scores, taking repeated measures within participants into account. Quality of the model: For the exact specifications of the quality of the model, see Table 5.

The intercept was significant (estimate = 1.963, *p* = 0.014). The significant predictors (fixed effects analysis) were general optimism, with a positive effect (estimate = 0.157, *p* = 0.042), and ESSI-D, with a negative effect (estimate = − 0.061, *p* = 0.030). The effect of time point was not significant (estimate = 0.215, *p* = 0.172). As previously shown in the interaction model, there is variability in the PHQ- 2 scores between participants that is not explained by fixed effects (variance = 0.559, SD = 0.748), which is also confirmed in the lagged predictor model. The other predictors did not have significant delayed effects on the PHQ- 2 score. For further details, see Table 6.

**Table 6:**
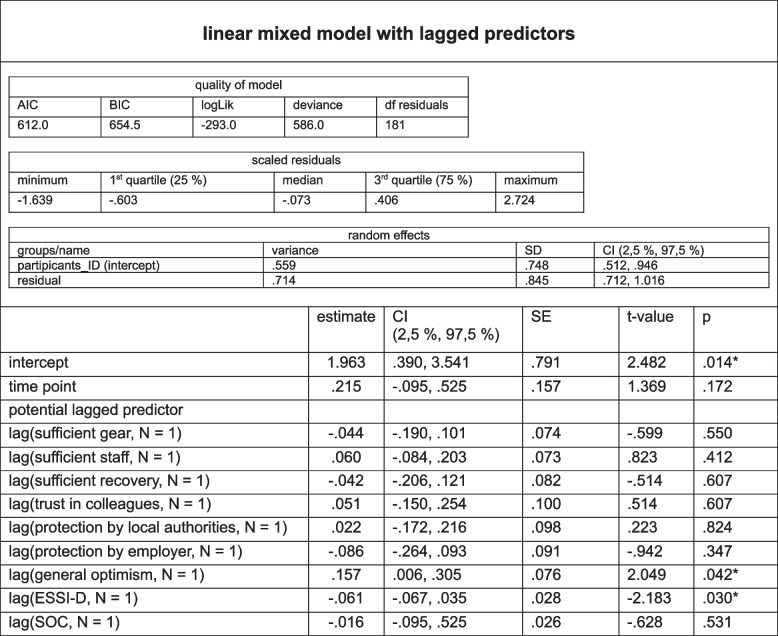
Linear mixed model with lagged predictors (external, individual, inter-relational resources) and the outcome variable severity of depressive symptoms (PHQ- 2 score) of psychotherapists at T1, T2, T3 and T4: longitudinal sample

adj. R² = adjusted R² (explained variance); significant *p*-values are marked: *: *p* ≤.050, **: *p* ≤.010, ***: *p* ≤.001; ESSI-D = ENRICHD Social Support Inventory; CI = confidence intervals; SD = standard deviation; df = degrees of freedom; logLik = log - likelihood; AIC = Akaike Information Criterion; BIC = Bayesian Information Criterion

## Discussion

*Key results:* The present survey preliminarily investigated the mental health of a total of 1733 psychotherapists within more than two years at four time points of the COVID- 19 pandemic in Germany, with a special focus on the impact of external, individual and inter-relational resources on perceived depression. Compared to HCW of mixed professions, psychotherapists reported lower mental distress, which increased throughout the pandemic. Linear modelling revealed a substantial protective effect of individual and inter-relational resources throughout the pandemic in psychotherapists.

### Mental distress of psychotherapists compared with HCW of other professions

Psychotherapists had a healthier start into the pandemic, with lower rates of depression than HCW, with prevalence rates of 14% versus 20.7%. One explanation could be a general reduction in elective appointments of patients, staying away from hospitals, e.g. due to fear of infections. An increase in depression scores from T1 to T2 stabilised after a decline at T3, two years after the pandemic. One can only be speculate, that for example, improved access to vaccination, adaptation to the crisis conditions and, at least in part, a perceived global control of the crisis contributed to this stabilisation. So far, there is little data on the psychological distress of psychotherapists during the pandemic. However pointing in a similar direction, a recent Austrian cross-sectional study on the mental health in 500 psychotherapists revealed lower rates of mental distress compared with the general population [35]. Other very specific longitudinal data from therapists suggested an increase in individual resources such as self-confidence and post-traumatic growth, shortly after the outbreak of the pandemic, which stabilised during the first months of the pandemic [31]. These findings may also help explain the healthier start into the pandemic. However, psychotherapists are exposed to the same general challenges of an ongoing crisis, such as caring for family members, restricted social contacts, as well as occupational strain such as fear of getting infected and uncertainty about the course of the pandemic. In terms of resources, psychotherapists in particular are experts in the field of functional coping, self-care and mental hygiene in the face of acute and ongoing stressors [34]. At the same time, the results of our investigation revealed stable resources such as optimism, SOC, social support, which are overall comparable to the resources of HCW of mixed professions. However, perceived support from the local authorities at all times and support from the employer at T2 were higher for psychotherapists than for HCW of mixed professions.

Aspects to be considered relate to specific key elements of the working practice of psychotherapists and their teams. These include frequent inter- and supervisions and team briefings, which are thought to help reduce long-term mental distress by promoting personal and team resilience. In line, a small longitudinal survey among psychotherapists suggested a well-established resilience of psychotherapists at the onset of the COVID- 19 pandemic [31]. However, the perception of sufficient recovery and sufficient staff decreased as the pandemic progressed, leading to the assumption that psychotherapists were becoming increasingly exhausted over time. In support of this theory, psychotherapists are known to overestimate their competence and well-being, and tend to continue working despite burnout or compassion fatigue [31]. Longitudinally, as the interaction effect of sufficient recovery during spare time on depression was negative, it might be suggested that changes in recovery patterns over time might be associated with later changes in depressive symptoms among psychotherapists. Previous studies have found that psychotherapists are at an increased risk of emotional exhaustion and fatigue [20, 23]. The above-mentioned and previously known overestimation of their competence and well-being, and the tendency to continue working despite burnout or compassion fatigue [31], might play an important role here. These findings are supported by the results from a mixed sample of HCW in clinics, suggesting the inability to recover as a risk factor for mental distress [13]. The opportunity to refresh between shifts is assumed to contribute to mental stability. Psychotherapists did not report a lack of staff at the beginning, which we suspected to be the origin of insufficient recovery. Instead, one reason for insufficient recovery could be the uncertainties and unpredictability of the spread of the virus. As a result, ruminative thinking and anxiety and/or additional challenges such as home schooling and home office might have led to a lack of recreation [13]. This explanation is supported by our results, as it could be suggested, that these challenges might have eventually diminished. An association with insufficient recovery and therefore an impact on depression might have been replaced by other effects on exhaustion or aspects, not covered within our study. A negative interaction effect of sufficient recovery during spare time with time on depression could also indicate adaptation to the crisis through other resources, such as SOC or other meaningful resources, not captured by our study. Our results could also suggest that it might be particularly beneficial to prioritise a sufficient recovery of psychotherapists at the onset of future crises. However, as the main effect of sufficient recovery was not significant, these results should be interpreted with caution. The observed interaction with time suggests that the effect may only be relevant at certain time points or may be influenced by other variables not captured in our analysis. Nevertheless, since the interaction effect was significant and negative, our results suggest that the influence of recovery during spare time is stronger at earlier time points, but may diminish over time. Given the context and content of the study, it seems reasonable to assume that this decreasing influence may explain why the main effects are not significant; the initially significant effects may have dissipated, reducing the overall influence when averaged over the entire study period. Further research is warranted to disentangle this complex relationship and to explore potential factors, not included in this study.

The sense of coherence was a stable protective resource against depression during the pandemic and predicted lower depression scores of longitudinally. Similarly, in a review of 458 studies, sense of coherence was found to be a major predictor of mental health in the general population. Higher levels of sense of coherence were negatively related to depression, anxiety and post-traumatic stress disorder [52]. In HCW of different professions, a higher sense of coherence was linked to fewer mental health problems [53–56]. Within the COVID- 19 pandemic, sense of coherence was identified as an important stress-buffering resource among HCW at the beginning of the crisis [13, 49]. However, the current study adds a longitudinal perspective, whereas our results suggest that a higher sense of coherence at the beginning of the pandemic may predict lower depression later on. The sense of coherence, which is thought to be inversely related to self-doubt and uncertainty, as previously described in the literature [83], remained stable throughout the COVID- 19 pandemic. During this time, psychotherapists experienced moderate levels of professional self-doubt, higher than in the pre-pandemic period. However, this self-doubt decreased over time, thus showing a resilient trajectory [31]. Similarly, the sense of coherence was stable and collaterally able to reduce mental distress. Furthermore, differences in the sense of coherence may indicate differences between psychotherapists and HCW of mixed professions in the general perception of the work environment and an entangled systemic perspective in times of crisis, contributing to differences in the perception of security and coherence. Both, the causal relationships of long-term burden and the influence of the sense of coherence merit further thorough investigation.

Furthermore, general optimism has been suggested to be protective against depression. Several studies have found positive associations between optimism and physical and mental well-being [44], as well as negative associations with depression, suicide, and feelings of helplessness [45, 47, 48]. Consistent with our results for psychotherapists, optimism was found to be protective against distress in HCW, in a previous large cross-sectional analysis by our work group [19]. Although optimism is a relatively stable personality trait [46], it can change over time and be increased by interventions [84, 85]. For psychotherapists, our data suggest a promising stability of optimism during the crisis. Interestingly, in our exploratory approach with lagged predictors, high optimism at the beginning of the crisis tended not to protect against depression throughout the pandemic. These results are very compelling, but need to be interpreted with caution due to the small sample size and the number of missing observations. They may suggest that psychotherapists with high optimism at the beginning (with a positive effect on their current depression) might be at relative risk of higher depression later in the pandemic as the protective effect diminishes longitudinally (also shown by our results), making them relatively more vulnerable.

On more than one dimension, inter-relational resources showed a significant effect on the mental health of psychotherapists, consistent with several previous findings from studies of HCW [13, 43, 49, 67].

Social support had a significant negative effect on subsequent PHQ- 2 scores, suggesting that greater social support earlier in the pandemic is predictive of lower depressive symptoms later. Items of the ESSI-D included for instance the capability of having someone to listen to, when conversation is needed, having someone for advice if problems occur, having someone for love and affection, having someone to rely on for emotional support, or having as much contact as needed with a close person. A large systematic review identified clear communication and high-quality of social support as protective factors for mental health in the general population during the COVID- 19 pandemic ( [61–63]; for a systematic review: [47]). Our results underscore the protective role and suggest a rather dominant role of social support on the mental health of psychotherapists during the pandemic. In line with our findings, social support was found to be strongly associated with lower job strain and better health outcomes in HCW during the pandemic [19, 60–63]. On the opposite side, years before the pandemic, lack of social support [64] or perceived loneliness have been identified as significant risk factors for mortality, which are equal to smoking, obesity and not exercising [65]. HCW want clear assurances that their organisation will support them and their family, listen to their concerns, do everything possible to protect and prevent them from COVID- 19 infection, and be assured of support on all fronts, both, medical and social [39]. Previous research has identified „talking to a friend or colleague at work “ as the most effective coping mechanism cited by clinical psychologists and other HCW [83]. Discussing difficult clients with peers and colleagues also helped family therapists cope with the daily stressors of practicing [68]. The increasing demands of a functioning team during the pandemic situation, leading to mental distress due to a lack of trust in one's own work group, have been noted previously [13]. However, the current findings also show an increase in solidarity and team cohesion in times of crisis [86, 87]. In addition, our study results revealed a stability of consent with trust in colleagues within the cohort of psychotherapists, adding a longitudinal perspective to current research results, at least for psychotherapists.

The study results showed a negative interaction effect of trust in colleagues with time on depression, which may also point to habituation to the crisis with the help of other resources, such as SOC, as well as other meaningful resources with a meaningful impact (newly developed or re-activated) that were not captured by our investigation. The observed interaction with trust in colleagues and time suggests that the effect may vary across time points or be moderated by other variables not captured in our analysis. However, as the effect was significant and negative, this may indicate that the influence is stronger in the early time points, but possibly diminishes over time. This possible diminishing influence could help explain why the main effects are not significant; initially significant effects may have dissipated over the study period, reducing the overall effect when averaged across all time points.

However, this complex interplay cannot be fully disentangled completely. Our results should be interpreted with caution due to an insignificant main effect of trust in colleagues. Nevertheless, given the context and the known importance of social support, it seems reasonable to assume that investing in a trustworthy and supportive team of colleagues—e.g. by avoiding changes in teams—could be beneficial, particularly at the onset of future crises, a notion supported by the general positive effects of social support.

### Strengths and limitations

To the best of our knowledge, the present survey preliminarily investigated the mental health of the largest overall sample of psychotherapists at four different time points over more than two years of the COVID- 19 pandemic in Germany. A wide range of investigated variables were assessed with respect to resources for mental distress and compared with a large relevant comparison sample of HCW of mixed professions. Although, an additional comparison sample from the general population was not assessed, our study included a longitudinal investigation of a smaller cohort of psychotherapists. The small sample size of the longitudinal sample, with most participants only participating at two time points, which resulted in many missing data in the analyses of all four time points, is a clear weakness. Although the longitudinal participants did not differ on any of the sociodemographic variables across the four time points in relation to the number of times they had participated, the sample size was small, so the results should at best be interpreted within the sociodemographic and work context examined.

The generalisability of the findings is limited in several ways and bias in several directions cannot be completely ruled out. As the survey design is mostly cross-sectional, causal conclusions cannot be drawn. Due to the method of data collection, a possible selection bias of the sample has to be considered. The data were self-reported and therefore cannot be objectively verified. Anonymous self-reporting character was required for ethical reasons, in order to protect privacy. Another limitation is the voluntary nature of our study, which may lead to response bias. The design allowed only a self-selected sample within the targeted cohort. Although both dropout analyses showed no fundamental differences with respect to sociodemographic, resource and primary outcome measures, for certain factors (e.g. having children; protection by local authorities, working in university clinics; PHQ- 2) few differences with very low effect sizes could be identified, but no effects in the analyses of interest in the present study. Given the significant dropout rate of over 50%, psychotherapists with higher levels of psychological distress and with children may not have had the energy or time to participate more often. Thus, psychotherapists with children, psychotherapists with higher levels of mental distress or psychotherapists not working in university clinics may be underrepresented in the longitudinal sample, and high dropout rates may bias the results in a healthier direction. Moreover, although rather unlikely with an online questionnaire, a social desirability cannot be completely ruled out. People working in university clinics may have been more enthusiastic about taking part in a study conducted by their institution. It is possible that there is an association between place of work and trust in local authorities, e.g. because of government funding of university hospitals. As mentioned above, the sample may not be representative of all psychotherapists working in Germany, and more comprehensive analyses in terms of different sociodemographic and work setting aspects of psychotherapists are warranted. Larger longitudinal studies that attempt to establish causality in relation to the complex interplay between health-promoting resources and HCW psychological distress would be beneficial. In addition, for economic reasons, data were assessed using short versions of the original instruments, such as for depression, which may reduce reducing criterion validity. However, the PHQ- 2 is well-studied and widely used [70], and these limitations were necessary for this study to increase the economy and ease of use for HCW already challenged by the pandemic. Nevertheless, by presenting results at different time points and including a longitudinal sample, the study contributes to the knowledge of the mental health of psychotherapists during the pandemic and adds fundamental evidence to the research published to date.

*Conclusion and Implications:* The present study introduces some important new findings, including lower but increasing levels of depression symptoms among psychotherapists compared with other HCWs during the COVID- 19 pandemic. At the same time, general optimism, sense of coherence and social support were mostly comparable and stable. The results showed a stable protective effect of sense of coherence, a positive association between high social support and low depression, and a decreasing effect of optimism throughout the pandemic. Such findings of a lower distress and a good set of stable and protective resources among psychotherapists on the one hand invite a reconsideration of the role of psychotherapists during crises. Psychotherapists could support burdened colleagues from other professions through even more regular multidisciplinary team intervisions or balint groups or COVID- 19 decision making boards as psychotherapists seem to have the capability, resources and mental strength to engage in supportive services. On the other hand, the evidence of increased levels of depression among psychotherapists during the pandemic is a reminder that psychotherapists need to manage their resources carefully in order to protect their own mental health. Although regarded as experts in the identification, treatment and rehabilitation of mental distress, it has been suggested that many fail to assess and acknowledge their own feelings of distress, potentially resulting in harm to psychotherapists and their patients [88]. Suggested strategies to prevent burnout include regular supervision, support with ethical practices of therapy, continuing education and involvement in non-professional activities, as well as creating a work-family balance [89]. One finding warrants further attention in future studies: psychotherapists with high optimism and a positive effect on their current depression at the beginning might be at relative risk for higher depression later in the pandemic, as the protective effect diminishes longitudinally, making them more vulnerable. This finding may reflect a certain vulnerability of optimistic psychotherapists and raise research questions about causal relationships. The demonstrated stable protective effect of the sense of coherence, a positive association of high social support and low depression throughout the pandemic, should encourage professional associations and policy makers to strengthen the sense of coherence in particular, and to promote social support to improve the coping of psychotherapists in future crises.

## Supplementary Information

Supplementary Material 1.

## Data Availability

The datasets used and/or analysed during the current study are available from the corresponding author upon reasonable request.

## Abbreviations

COVID- 19: COrona VIrus Disease-2019
CS: Comparison sample
ENRICHD: Enhancing Recovery in Coronary Heart Disease Patients
ESSI-D: ENRICHD Social Support Inventory-D
HCW: Health Care Workers
PHQ: Patient Health Questionnaire
PT: Psychotherapists
SOC: Sense of coherence
