# Supplementary material for: Course of mental distress among psychotherapists throughout two years of the COVID- 19 pandemic: individual and inter-relational resources make a difference—cross-sectional and longitudinal results of the VOICE study
Source: BMC Psychiatry. 2025 May 6;25:456. doi: 10.1186/s12888-025-06867-4 (PMC12057149; doi:10.1186/s12888-025-06867-4)
Supplement: Supplementary file 1 — Supplementary Material 1. [file 12888_2025_6867_MOESM1_ESM.docx]

**Supplementary material:**

***Table 1S:* Description of the longitudinal study sample**

| **variable of interest**  total of T1, T2, T3 and T4 | **psychotherapists**  n (%)  252 (100)  participations number:  2: n = 194, 3: n = 45, 4: n = 13 |
| --- | --- |
| **gender** male  female  diverse  missing | 39 (15.5)  213 (85.4)  0 (0)  0 (0) |
| **age group** 18 - 30  (years) 31 - 40  41 - 50  51 - 60  61 - 70  > 70  missing | 52 (20.6)  76 (30.2)  59 (23.4)  58 (23.0)  6 (2.4)  1 (.4)  0 (0) |
| **living alone** yes  no  missing | 43 (17.1)  209 (82.9)  0 (0) |
|  |  |
| **having children**  yes, own household  yes, different household  no  missing | 111 (44.0)  18 (7.1)  123 (48.8)  0 (0) |
| **workplace**  university clinic  clinic  ambulatory health care center  outpatient/practice  missing | 118 (46.8)  49 (19.4)  5 (2.0)  21 (8.3)  59 (23.4) |
| **work experience**  < 3 years  3 - 6 years  > 6 years  not in direct patient care  missing | 47 (18.7)  43 (17.1)  156 (61.9)  4 (1.6)  2 (.8) |
| **change of** yes  **department**  no  missing | 27 (10.7)  224 (88.9)  1 (.4) |
| **contact with COVID-19**  yes  no  missing | 27 (10.7)  224 (88.9)  6 (.4) |
| **participation of psychotherapists (PT) (time points)** | **N** |
| **PT participated at two time points** | 194 |
| **T1 + T2** (not, T3, T4) | 89 |
| **T1 + T3** (not, T2, T4) | 20 |
| **T1 + T4** (not T2, not T3) | 12 |
| **T2 + T3** (not T1, not T4) | 24 |
| **T2 + T4** (not T1, not T3) | 23 |
| **T3 + T4** (not T1, not T2) | 26 |
| **PT participated at three time points** | 45 |
| **T1 + T2 + T3** (not T4) | 13 |
| **T1 + T2 + T4** (not T3) | 6 |
| **T1 + T3 + T4** (not T2) | 8 |
| **T2 + T3 + T4** (not T1) | 18 |
| **PT participated at four time points** | 13 |
| **T1 + T2 + T3 + T4** | 13 |
| total of participants at least participated in twice | 252 |

***Table* 2S: Content of assessed work- and COVID-19 related variables**

| **variable**  **abbreviation** | **content**  **assessed work-related variable** |
| --- | --- |
|  | |
| **work. cond. 1 *** | There is sufficient protective gear. |
| **work. cond. 4** | There is sufficient staff. |
| **work. cond. 5** | I can recover sufficiently during spare time. |
| **work. cond. 6** | I can trust in my colleagues during difficult times at work. |
| **COVID-19 related problems** | |
| **COVProb_5** | I felt protected by local authorities. |
| **COVProb_6** | I felt protected by my employer. |
| COVProb = COVID-19 related risks and resources; work. cond. = work-related risks and resources *: not assessed at time point 3 and time point 4 | |

***Table 3S:* Drop out analysis: comparison of cross-sectional and longitudinal participants at T1**

| **variable of interest** | **psychotherapists (PT)**  **at T1**  **N=689** | | **statistics**  **PT /CS**  χ2-test  χ2, Cramer`s V, p |
| --- | --- | --- | --- |
|  | one participation  N (T1) = 528 | more than one participation  N (T1) = 161 |  |
|  | cross-sectional sample | longitudinal sample |  |
| **gender** male  female  diverse | 138 (26.1)  387 (73.3)  3 (.6) | 25 (15.5)  136 (84.5)  0 (.0) | 8.814, .113, .012* |
| **age-group** 18-30  31-40  41-50  51-60  61-70  >70 | 68 (12.9)  105 (19.9)  129 (24.4)  160 (30.3)  62 (11.7)  4 (.8) | 32 (19.9)  40 (24.8)  34 (21.1)  49 (30.4)  5 (3.1)  1 (.6) | 15.673, .151, .008** |
| **living alone** yes  no | 110 (20.8)  418 (79.2) | 25 (15.5)  136 (84.5) | 2.204, .057, .138 |
| **having children**  yes, own household  yes, different household  no | 221 (41.9)  111 (21.0)  196 (37.1) | 71 (44.1)  15 (9.3)  75 (46.6) | 12.200, .133, .002** |
| **workplace**  university clinic  clinic  ambulatory health care center  practice | 124 (23.5)  120 (22.7)  5 (.9)  111 (21.0) | 70 (43.5)  30 (18.6)  19 (11.8)  3 (1.9) | 28.253, .203, <.001*** |
| **work experience**  < 3 years  3 - 6 years  > 6 years  not in direct patient care | 72 (13.6)  65 (12.3)  382 (72.3)  9 (1.7) | 28 (17.4)  24 (14.9)  106 (65.8)  3 (1.9) | 2.598, .061, .458 |
| **change of** yes  **department**  no | 65 (12.4)  460 (87.6) | 21 (13.1)  139 (86.9) | .062, .010, .804 |
| **contact with** yes  **COVID-19** no | 72 (13.7)  453 (86.3) | 13 (8.1)  147 (91.9) | 3.525, .072, .060 |
| **PHQ-2 rate** n (%) | 77 (15.7) | 14 (8.9) | 4.476, .083, .034* |
|  |  |  | **statistics PT/CS**  t-test  F, p, Cohen`s D |
| **PHQ-2 score**  mean (SD) n | 1.445 (1.371) 492 | 1.204 (1.131) 157 | 5.869, .028, 1.374 |
| **general optimism**  mean (SD) n | 5.045 (1.253) 487 | 5.252  (1.137) 155 | 1.023, .068, 1.226 |
| **SOC**  mean (SD) n | 15.635(3.367) 490 | 15.628, 3.215 .983 | 1.182, 3.331, .983 |
| **ESSI-D**  mean (SD) n | 20.852 (3.878) 492 | 21.527 (3.264) 156 | 3.338, 3.740, .050* |
|  |  |  | **statistics**  **PT /CS**  χ2-test  χ2, Cramer`s V, p |
| **sufficient protective gear**  **consent (%)** | 272 (54.7) | 88 (64.2) | 3.954, .079, .047 |
| **sufficient staff**  **consent (%)** | 286 (57.7) | 78 (56.9) | .023, .006, .879 |
| **sufficient recovery**  **consent (%)** | 212 (42.7) | 57 (41.6) | .048, .009, .826 |
| **trust in colleagues**  **consent (%)** | 345 (72.6) | 96 (71.6) | .051, .009, .821 |
| **protection by local authorities**  **consent (%)** | 253 (49.9)  507 | 102 (63,.7)  160 | 9.369, .119, .002** |
| **protection by employer**  **consent (%)** | 261 (51.5)  246 (48.5)  507 | 83 (51.9)  77 (48.1)  160 | .008, .003, .930 |
| PT = psychotherapists; significant p-values are marked: *: p ≤ .050, **: p ≤ .010, ***: p ≤ .001; Bonferroni correction: p ≤ .050/4 (.013) for 4 score variables with means (PHQ-2 score, general optimism, SOC and ESSI-D) and p ≤ .05/15 (.003) for 15 categorial variables: 8 sociodemographic variables + PHQ-2 rate + 6 resource variables; PHQ-2 = separate module of the PHQ-4 (Patient Health Questionnaire) assessing depression; cut off for PHQ-2-score for clinically relevant depression: ≥ 3; T1, T2, T3, T4 = time point 1, 2, 3 and 4 | | | |

***Table 4S:* Drop out analysis: comparison of sociodemographic variables of psychotherapists at all 4 time points in relation to their number of participations**

| **variable of interest** | **psychotherapists**  longitudinal sample | | | |
| --- | --- | --- | --- | --- |
|  | **T1** N=161 | **T2**  N=186 | **T3** N=122 | **T4** N=106 |
|  | χ2  number of participations  (2, 3 or 4)  Cramer`s V, p | χ2  number of participations  (2, 3 or 4)  Cramer`s V, p | χ2  number of participations  (2, 3 or 4)  Cramer`s V, p | χ2  number of participations  (2, 3 or 4)  Cramer`s V, p |
| **gender**  male, female, diverse | 1.091, .082, .580 | .581, .056, .748 | .783, .080, .676 | 2.111. .141, .348 |
| **age group**  18-30, 31-40, 41-50  51-60, 61-70, >70 | 3.916, .110, .951 | 10.184, .165, .425 | 8.162, .183, .418 | 11.276, .231, .187 |
| **living alone**  yes, no | 2.634, .128, .268 | 2.523, .116, .283 | 7.343, .245, .025 | .254, .049, .881 |
| **having children**  yes, own household  yes, different household  no | 1.688, .072, .793 | 1.389, .061, .846 | 2.603, .103, .626 | 8.519, .200, .074 |
| **workplace**  university clinic, clinic, ambulatory health care center, practice | 8.581, .163, .572 | 11.729, .251, .304 | 14.539, .244, .069 | 14.178, .259, .077 |
| **work experience**  < 3 years, 3-6 years, > 6 years, not in direct patient care | 2.331, .085, .887 | 13.337, .189, .038 | 2.865, .112, .581 | 3.656, .133, .455 |
| **change of** **department**  yes, no | 1.519, .098, .468 | 1.258, .082, .533 | .443, .060, .801 | 3.067, .170, .216 |
| **contact with COVID-19** yes, no | 1.359, .092, .507 | 1.648, .094, .439 | 4.688, .196, .096 | 1.813, .131, .404 |
|  | ANOVA  factor: number of participations (2, 3 ,4)  F, η_p_^2^, p | ANOVA  factor: number of participations (2, 3 ,4)  F, η_p_^2^, p | ANOVA  factor: number of participations (2, 3 ,4)  F, η_p_^2^, p | ANOVA  factor: number of participations (2, 3 ,4)  F, η_p_^2^, p |
| **PHQ-2 score (**mean) | .  518, .007, .597 | .408, .004, .66 | .309, .005, .735 | .180, .004, .835 |
| **general optimism (**mean) | 2.174, .028, .115 | .322, .004, .725 | 3.96, .007, .674 | .286, .006, .752 |
| **SOC (**mean) | .056, .001, .946 | .876, .010, .418 | .458, .008, .633 | .638, .013, .530 |
| **ESSI-D (**mean) | 1.573, .020, .211 | .  163, .002, .850 | .252, .004, .778 | .585, .012, .559 |
|  | χ2  2, 3 or 4 participations  Cramer`s V, p | χ2  2, 3 or 4 participations  Cramer`s V, p | χ2  2, 3 or 4 participations  Cramer`s V, p | χ2  2, 3 or 4 participations  Cramer`s V, p |
| **sufficient protective gear**  **consent (%)** | 3.138, .151, .208 | .930, .081, .628 | **** | **** |
| **sufficient staff**  **consent (%)** | 1.748, .113, .417 | .840, .077, .657 | .115, .031, .944 | 1.224, .108, .542 |
| **sufficient recovery**  **consent (%)** | 5.151, .193, .076 | .  267, .043, .875 | 2.403, .140, .301 | 2.954, .168, .228 |
| **trust in colleagues**  **consent (%)** | 2.661, .140, .264 | 3.343, .153, .188 | 1.327, .103, .515 | 2.742, .162, .254 |
| **protection by local authorities**  **consent (%)** | 3.807, .155, .149 | 4.033, .148, .133 | 3.720, .175, .156 | 1.237, .114, 539 |
| **protection by employer**  **consent (%)** | .581, .060, .748 | .440, .049, .803 | .997, .090, .608 | .716, .086, .699 |
| PT = psychotherapists; significant p-values are marked: *: p ≤ .050, **: p ≤ .010, ***: p ≤ .001; Bonferroni correction: p ≤ .050/4 = .013 for 4 score variables with means (PHQ-2 score, general optimism, SOC and ESSI-D) and p ≤ .050/15 (003) for 15 categorial variables: 8 sociodemographic variables + PHQ-2 rate + 6 resource variables; PHQ-2 = separate module of the PHQ-4 (Patient Health Questionnaire) assessing depression; cut off for PHQ-2-score for clinically relevant depression: ≥ 3; T1, T2, T3, T4 = time point 1, 2, 3 and 4; ****: not assessed at this time point | | | | |

|  |
| --- |

***Table 5S:* Transparency of model selection process - linear model: cross-sectional samples**

| **At first a LMM** (linear mixed model fit by maximum likelihood) **with time point as random effect was conducted.** | | | | | |
| --- | --- | --- | --- | --- | --- |
| **model equation** | lmer( PHQ-2 ~ time point * (sufficient gear + sufficient staff + sufficient recovery + trust in colleagues + protection by local authorities + protection by the employer + general optimism + ESSI-D + SOC) + (1 \| time point), *data = data, REML = FALSE* | | | | |
| **model fit** | **AIC** | **BIC** | **logLik** | **deviance** | **df residuals** |
|  | 3014.1 | 3122.9 | 1485.0 | 2970.1 | 1015 |
| **scaled residuals** | **minimum** | 1^st^ quartile (25 %) | **median** | 3^rd^ quartile (75 %) | **maximum** |
|  | -4.054 | -.668 | -.056 | .568 | 4.584 |
| **random effects** | **groups name** | **variance** | | **standard deviation** | |
|  | **time point** | .000 |  | .000 |  |
|  | **residual** | 1.027 |  | 1.013 |  |
|  | number of observations: 1037, groups: time point, 2 | | | | |
|  | | | | | |
| **rationale for using a linear model**  The decision to use a linear model (LM) was based on initial analyses using a linear mixed model (LMM) under the same conditions, which included time point as a random effect. The LMM showed negligible variance for the random effect of time point (variance = .000, SD = .000), indicating no significant impact on the dependent variable. This suggested that modeling time as a random effect did not improve variance explanation. Therefore, we opted for a linear model without random effects, focusing on fixed predictors and their interactions with time. This approach allowed us to directly examine the significance of key predictors and their interactions across all time points, aligning the model with the cross-sectional nature of the data and our research objectives. | | | | | |
| **Based on the decision not to include time point as random effect, we opted for a linear model fitted by maximum likelihood.** | | | | | |
| **model equation** | lm( PHQ-2 ~ time point * (sufficient gear + sufficient staff + sufficient recovery + trust in colleagues + protection by local authorities + protection by the employer + general optimism + ESSI-D + SOC), *data = data, REML = FALSE* | | | | |
| **model fit** | **multiple R-squared** | | **adjusted R-squared** | | |
|  | .419 | | .409 | | |
| These values point toward an explanation of variance of 40.9 % in the dependent model. This is a moderate degree of explanation, pointing towards an acceptable adaption of the model of the data. | | | | | |
| **residuals** | minimum | 1^st^ quartile (25 %) | **median** | 3^rd^ quartile (75 %) | **maximum** |
|  | -4.107 | -.677 | -.057 | .575 | 4.645 |
| **linear model** | | | | | |
| **variable of interest** | **estimate** | **CI**  **2,5 %, 97,5 %** | **SE** | **t-value** | **p** |
| **potential main effects** | | | | | |
| intercept | 6.468 | 5.055, 7.882 | .720 | 8.982 | <.001*** |
| time point | .038 | -.890, .967 | .473 | .081 | .935 |
| sufficient equipment | -.120 | -.291, .052 | .088 | -1.365 | .172 |
| sufficient staff | .001 | -.157, .159 | .081 | .012 | .990 |
| sufficient recovery | .006 | -.175, .188 | .092 | .068 | .946 |
| trust in colleagues | .127 | -.082, .337 | .107 | 1.190 | .234 |
| protection by local authorities | -.097 | -.317, .123 | .112 | -.862 | .389 |
| protection by employer | -.048 | -.253, .157 | .104 | -.461 | .645 |
| general optimism | -.134 | -.309, .041 | .089 | -1.503 | .133 |
| ESSI-D | -.025 | -.083, .032 | .029 | -.861 | .390 |
| SOC | -.227 | -.294, -.161 | .034 | -6.704 | <.001*** |
| **potential interaction effects** | | | | | |
| time point * sufficient equipment | .070 | -.051, .190 | .061 | 1.137 | .256 |
| time point * sufficient staff | .009 | -.098, .115 | .054 | .161 | .872 |
| time point * sufficient recovery | -.143 | -.263, -.024 | .061 | -2.354 | .019* |
| timepoint * trust in colleagues | -.147 | -.284, -.010 | .070 | -2.105 | .036* |
| time point * trust in local authorities | .044 | -.100, .188 | .073 | .599 | .549 |
| time point * trust in employer | -.003 | -.133, .127 | .066 | -.043 | .965 |
| time point * general optimism | .043 | -.071, .158 | .058 | .744 | .457 |
| time point * ESSI-D | -.015 | -.052, .023 | .019 | -.760 | .448 |
| time point * SOC | .046 | .004, .089 | .021 | 2.135 | .033* |
| AIC = Akaike information criterion; BIC = Bayesian Information Criterion; df = degrees of freedom; CI = confidence intervals; logLik = log-likelihood, PHQ-2 = separate module of the PHQ-4 = (Patient Health Questionnaire) assessing depression; cut off (PHQ-2) for clinically relevant depression: ≥ 3, SOC = sense of coherence; ESSI-D = ENRICHD Social Support Inventory | | | | | |
| **statement multicollinearity** | | | | | |
| **No significant multicollinearity** was existent in the LM: despite the relatively high GVIF (**generalized variance inflation factor)**-values, the corrected values GVIF^(1/(2 * df)) were all below 5, indicating that no problematic multicollinearity was existing in the present model. These values are in general acceptable, indicating that the estimated coefficients very not strongly distorted through multicollinearity.  Adequate modeling: Since the GVIF^(1/(2 * df))-values all lie within an acceptable range, it can be suggested that interactions in the model are well modeled and there is no „inflation“ of the variance, potentially impairing the interpretation of the results. | | | | | |
| **generalized variance inflation factor (GVIF)-values for the predictors of the model/ interpretation of the GVIF-values** | | | | | |
| 1.**GVIF: time point:** GVIF = 1.000; indicating no multicollinearity present, which was expected, since time points was treated as a categorial variable, which is in interaction with the other predictors. **Sufficient protective gear:** GVIF = 85.864; this value is relatively high, however the GVIF^(1/(2 * df) - value of 2.100 shows that the potential inflation of variance is relatively low, if the degrees of freedom are considered. **Other predictors:** The GVIF-values of the other predictors (e. g. sufficient staff, sufficient recovery) were between 72 and 110, but GVIF^(1/(2*df))-values were between 2.04 and 2.19, which is suggested to be acceptable. | | | | | |
| **2. Interpretation of the GVIF^(1/(2 * df)) values:** These values represent the factor of inflation on the level of the individual predictors, corrected by the count of degrees of freedom. This means, even relatively high GVIF values among single predictors, do not need to be considered problematic**.** | | | | | |
| **3. Interactions:** Results show an interaction of timepoint with every predictor, however, the GVIF-values remains low, time point is used as a basefactor, with is combined with all other predictors. For the other predictors each of them interacts with timepoint and is influenced by the other predictors (see other predictors) | | | | | |
| AIC = Akaike information criterion; BIC = Bayesian Information Criterion; df = degrees of freedom; CI = confidence intervals, logLik = log-likelihood | | | | | |

***Table 6S:* Transparency of model selection process- LMMs: longitudinal sample**

| **First, we opted for a base model with only the intercept as random effect linear mixed model fit by maximum likelihood ['lmerMod'].** | | | | | |
| --- | --- | --- | --- | --- | --- |
| **model equation** | *lmer(PHQ-2 ~ time point + sufficient gear + sufficient staff + sufficient recovery + trust in colleagues + protection by local authorities + protection by employer + general optimism + ESSI-D + SOC + (1 \| participant_ID), data = data, REML = FALSE* | | | | |
| **model fit** | AIC | BIC | logLik | deviance | df. resid. |
|  | 752.7 | 799.5 | 363.4 | 726.7 | 258 |
| scaled residuals | **minimum** | 1^st^ quartile (25 %) | **median** | 3^rd^ quartile (75 %) | **maximum** |
|  | 2.278 | -0.560 | -0.099 | 0.575 | 2.928 |
| random effects | | | | | |
| groups  name | **variance** |  | **standard deviation** |  |  |
| participant_ID  (intercept) | 0.184 |  | 0.428 |  |  |
| residual | 0.686 |  | 0.828 |  |  |
| number of observations: 271, groups: participant_ID, 175 | **271** | groups: participant_ID, | **175** |  |  |
| **VIF values (multicollinearity)** | **time point** | **sufficient gear** | **sufficient staff** | **sufficient recovery** | **trust in colleagues** |
|  | 1.255 | 1.220 | 1.209 | 1.292 | 1.208 |
|  | **protection by authorities** | **protection by employer** | **general optimism** | **ESSI-D** | **SOC** |
|  | 1.335 | 1.382 | 1.130 | 1.158 | 1.196 |
| AIC = Akaike information criterion; BIC = Bayesian Information Criterion; df = degrees of freedom; CI = confidence intervals, logLik = log-likelihood; VIF = variance inflation factor | | | | | |
| **ICC-Values were calculated to reflect if random effects would be needed.** | | | | | |
|  | **time point** | **sufficient gear** | **sufficient Staff** | **sufficient recovery** | **trust in colleagues** |
|  | 0.434 | 0.079 | 0.307 | 0.491 | 0.473 |
|  | **protection by authorities** | **protection by employer** | **general optimism** | **ESSI-D** | **SOC** |
|  | 0.335 | 0.374 | 0.718 | 0.729 | 0.602 |
| ICC (intraclass correlation coefficient) -values indicated, that the variability within the predictor was mainly explained by differences between the participants, which means predictors for each participant are relatively stable, while differences between the participants are pronounced. Such a high degree of variance between the participants suggests a rather subordinate role of the time point and a rather dominant role of the participant_ID. These results could suggest that a simplier model, with only one random intercept could also function very well. | | | | | |
| Given the low ICC for “sufficient gear”, we initially considered modeling it as a random effect in our linear mixed-effects model (LMM). The low ICC suggested that most of the variability in “sufficient gear” was due to changes within participants over time rather than differences between participants, indicating a time-variant effect. This would typically support the inclusion of a random effect to capture within-subject variability across time points. However, because “sufficient gear” was only assessed at time points 1 and 2 out of 4, the random effect model of this variable led to instability and a loss of statistical power. One main reason, this effect is so time dependent is the assessment only at T1 and T2. The limited data points, combined with the low ICC, meant that modeling it as a random effect did not improve the robustness of the model. Instead, it introduced complexity without providing additional explanatory power. Therefore, we decided to treat “sufficient gear” as a fixed effect, which resulted in a more stable and powerful model and more reliable parameter estimates. This approach better aligned with the structure of our data, ensuring greater stability and interpretability in our findings.  Excluding ‘sufficient staff’ (assessed only at T1 and T2) did not substantially improve the model, but it significantly reduced the explained variance and did not alter the significance of the remaining predictors and their relationships with time points | | | | | |
| **In the next step we opted to analyze the predictors influence of the PHQ-2 score at different time points (interactions between predictors and time points) -> model with interactions.** | | | | | |
| (time point * predictors): allowing the model to estimate different effects of the predictors on PHQ-2 for the different time points (time point one automatically is reference value) | | | | | |
| linear mixed model fit by maximum likelihood | ['lmerMod'] Formula: PHQ-2 ~ time point * (sufficient gear + sufficient staff + sufficient recovery + trust in colleagues + protection by local authorities + protection by employer + generel optimism + ESSI-D + SOC) + (1 \| participant_ID) *data = data_lag, REML = FALSE* | | | | |
| **LMM fit** | **AIC** | **BIC** | **logLik** | **deviance** | **df residuals** |
|  | 756.8 | 836.1 | -356.4 | 712.8 | 249 |
| scaled residuals | minimum | 1^st^ quartile (25 %) | median | 3^rd^ quartile (75 %) | maximum |
|  | -2.041 | .574 | -.048 | .544 | 2.747 |
| random effects |  |  |  |  |  |
| groups name | variance |  | standard deviation |  |  |
| participant_ID (Intercept) | .205 |  | .452 |  |  |
| residual | 0.626 |  | .791 |  |  |
| number of observations | 271 | groups: participant_ID, 175 |  |  |  |
| **linear mixed-effects model with main and interaction effects** | | | | | |
| **variable of interest** | **estimate** | **CI**  2.5 %, 97.5 % | **SE** | **t-value** | **p** |
| intercept | 5.337 | 2.478, 8.227 | 1.452 | 3.676 | <.001*** |
| time point | .323 | .1.545, 2.172 | .940 | .344 | .731 |
| sufficient gear | -.010 | -.316, .299 | .156 | -.058 | .954 |
| sufficient staff | .254 | -.047, .554 | .152 | 1.669 | .096 |
| sufficient recovery | -.015 | -.351, .324 | .171 | -.086 | .932 |
| trust in colleagues | -.091 | -.467, .286 | .191 | -.474 | .636 |
| protection by local authorities | -.006 | -.413, .407 | .206 | -.028 | .978 |
| protection by employer | .006 | -.380, .387 | .192 | .033 | .974 |
| general optimism | -.473 | -.794, -.153 | .163 | -2.911 | .004** |
| ESSI-D | -.011 | -.120, .097 | .055 | -.205 | .838 |
| SOC | -.119 |  | .058 | -2.069 | .040* |
| **potential interaction effects** | | | | | |
| time point * sufficient gear | .073 | -.127, .274 | .102 | .721 | .471 |
| time point * sufficient staff | -.169 | -.358, .021 | .096 | -1.767 | .078 |
| time point * sufficient recovery | -.174 | -.388, .037 | .107 | -1.632 | .104 |
| time point * trust in colleagues | .130 | -.121, .379 | .127 | 1.022 | .308 |
| time point * prot. by local authorities | .065 | -.189, .317 | .128 | .508 | .611 |
| time point * protection by employer | -.108 | -.344, .130 | .120 | -.906 | .365 |
| time point * general optimism | .227 | .034, .420 | .098 | 2.314 | .021* |
| time point * ESSI-D | -.031 | -.100, .038 | .035 | -.889 | .375 |
| time point * SOC | -.019 | -.086, .049 | .034 | -.561 | .575 |
| AIC = Akaike information criterion; BIC = Bayesian Information Criterion; df = degrees of freedom; CI = confidence intervals, logLik = log-likelihood; PHQ-2 = separate module of the PHQ-4 = (Patient Health Questionnaire) assessing depression; cut off (PHQ-2) for clinically relevant depression: ≥ 3, SOC = sense of coherence; ESSI-D = ENRICHD Social Support Inventory | | | | | |
| **We additionally opted for an exploratory approach with a LMM with lagged predictors.** | | | | | |
| Specifically with the question on an impact of the value of the predictors at earlier time points on PHQ-2 at later time points | | | | | |
| **model equation** | lmer( PHQ-2 ~ lag(sufficient gear, n = 1) + lag(sufficient staff, n = 1) + lag(sufficient recovery, n = 1) + lag(trust in colleagues, n = 1) + lag(protection by local authorities, n = 1) + lag(protection by employer, n = 1) + lag(general optimism, n = 1) + lag(ESSI-D, n = 1) + lag(SOC, n = 1) + time point + (1 \| participant_ID), data = REML = FALSE) | | | | |
| **LMM fit** | **AIC** | **BIC** | **logLik** | **deviance** | **df residuals** |
|  | 612.0 | 654.5 | 293.0 | 586.0 | 181 |
| scaled residuals | minimum | 1^st^ quartile (25 %) | median | 3^rd^ quartile (75 %) | max |
|  | -1.639 | -0.603 | -0.073 | 0.406 | 2.724 |
| random effects | | | | | |
| groups name | variance |  | standard deviation |  |  |
| participant_ID  (intercept) | .559 |  | .748 |  |  |
| residual | .714 |  | .845 |  |  |
| number of observations | 194 | groups: participant_ID | 141 |  |  |
| **linear mixed model with lagged predictors** | | | | | |
|  | **estimate**  **CI 95 %** | **CI**  2.5 %, 97.5 % | **SE** | **t-value** | **p** |
| intercept | 1.963 | .390, 3.541 | .791 | 2.482 | .014* |
| time point | .215 | -.095, .525 | .157 | 1.369 | .172 |
| **potential lagged predictor** | | | | | |
| lag(sufficient gear, n=1) | -.044 | -.190, .101 | .074 | -.599 | .550 |
| lag(sufficient staff, n=1) | .060 | -.084, .203 | .073 | .823 | .412 |
| lag(sufficient recovery, n=1) | -.042 | -.206, .121 | .082 | .-514 | .607 |
| lag(trust in colleagues, n=1) | .051 | -.150, .254 | .100 | .514 | .607 |
| lag(protection by local authorities, n=1) | .022 | -.172, .216 | ,098 | .223 | .824 |
| lag(protection by employer, n=1) | -.086 | -.264, .093 | .091 | -.942 | .347 |
| lag(general optimism, n=1) | .157 | .006, .305 | .076 | 2.049 | .042* |
| lag(ESSI-D, n=1) | -.061 | -.067, .035 | .028 | -2.183 | .030* |
| lag(SOC, n=1) | -.016 | -.095, .525 | .026 | -.628 | .531 |
| In this study, we opted to implement two separate **linear mixed-effects models (LMMs)** to explore the effects of time-dependent predictors on depressive symptoms. The first LMM included interaction terms between the time points and various predictors to capture how the relationships between work conditions, psychological factors, and depressive symptoms evolved over time. This model was feasible given the available data, allowing us to thoroughly investigate the temporal dynamics and contextual influences on mental health outcomes.  However, when we attempted to extend this model by incorporating lagged predictor variables that represent previous states of the predictors to account for delayed effects on depressive symptoms the model became too complex relative to the number of observations. The inclusion of both interaction terms and lagged predictors led to convergence issues and model instability, primarily due to insufficient data to support the estimation of such a highly parameterized model. Therefore, we conducted an exploratory and supplementary analysis using a separate LMM with lagged predictors only, without interaction terms. This approach allowed us to explore the potential impact of previous predictor states on the current depressive symptoms, without overburdening the model. The decision to separate these analyses was driven by the need to balance model complexity with the reliability of parameter estimates, ensuring that each model could provide meaningful insights into the different dimensions of the data. | | | | | |
| **rationale for not conducting likelihood ratio tests** (LRTs)  Our study prioritized the significance of specific predictors and their interactions with time, based on their theoretical and empirical importance. While LRTs could have been used to optimize model fit, we chose not to conduct them to avoid excluding key predictors. Our focus was on examining these predictors’ contributions, even if it meant potentially sacrificing some model fit, to ensure that our findings remained aligned with our research objectives. | | | | | |
| AIC = Akaike information criterion; BIC = Bayesian Information Criterion; df = degrees of freedom, CI = confidence intervals, logLik = log-likelihood; PHQ-2 = separate module of the PHQ-4 = (Patient Health Questionnaire) assessing depression; cut off (PHQ-2) for clinically relevant depression: ≥ 3, SOC = sense of coherence; ESSI-D = ENRICHD Social Support Inventory; | | | | | |

***Table 7S:* Correlation analyses of sociodemographic variables of psychotherapists with outcome parameter PHQ-2 score at time point of interest (T1, T2, T3, T4)**

| **timepoint of interest** | **gender** | **age group** | **living alone** | **having children** | **work place** | **work experience** | **change of department** | **contact with COVID-19** |
| --- | --- | --- | --- | --- | --- | --- | --- | --- |
| **PHQ-2** r  **T1** p  n | .049  .215  649 | .022  .422  649 | -.054  .171  649 | .019  .621  649 | .055  .246  455 | -.010  .807  649 | -.058  .139  649 | .010  .793  649 |
| **PHQ-2**  r  **T2** p  n | .075  .073  567 | -.041  .327  567 | -.055  .191  567 | .059  .158  567 | .072  .130  450 | -.021  .615  567 | -.078  .065  567 | .051  .227  567 |
| **PHQ-2** r  **T3**  p  n | .122  .071  219 | -.077  .255  219 | -.061  .369  219 | .061  .369  219 | .059  .420  187 | -.088  .195  217 | -.122  .071  219 | -.030  .661  219 |
| **PHQ-2** r  **T4**  p  n | .006  .928  218 | -.041  .545  218 | -.052  .442  218 | .100  .142  218 | .018  .794  202 | -.078  .252  216 | -.102  .133  218 | -.082  .226  218 |
| **longitudinal sample** |  |  |  |  |  |  |  |  |
| **PHQ-2 r** | .090 | -.106 | -.034 | .055 | -.064 | -.150 | -.122 | .011 |
| **T1/T1 p** | .264 | .188 | .674 | .498 | .545 | .062 | .130 | .891 |
| **n** | 156 | 156 | 156 | 156 | 93 | 156 | 156 | 156 |
| **PHQ-2 r** | .012 | -.091 | -.056 | -.096 | -.016 | -.105 | -.047 | .117 |
| **T1/T2 p** | .899 | .326 | .546 | .298 | .850 | .256 | .613 | .207 |
| **n** | 119 | 119 | 119 | 119 | 144 | 118 | 118 | 118 |
| **PHQ-2 r** | .052 | -.132 | -.198 | .033 | .196 | .042 | -.203 | -.067 |
| **T1/T3 p** | .707 | .341 | .150 | .812 | .152 | .762 | .145 | .631 |
| **n** | 54 | 54 | 54 | 54 | 55 | 54 | 53 | 53 |
| **PHQ-2 r** | .022 | -.064 | .059 | .070 | -.149 | .059 | .039 | .039 |
| **T1/T4 p** | .896 | .697 | .723 | .672 | .330 | .720 | .814 | .814 |
| **n** | 39 | 39 | 39 | 39 | 45 | 39 | 39 | 39 |
| **PHQ-2 r** | .156 | -.035 | -.099 | .041 | .032 | -.112 | -.152 | .018 |
| **T2/T3 p** | .205 | .777 | .421 | .742 | .816 | .365 | .217 | .886 |
| **n** | 68 | 68 | 68 | 68 | 55 | 68 | 68 | 68 |
| **PHQ-2 r** | .029 | -.054 | -.097 | .041 | .014 | -.079 | .176 | .181 |
| **T2/T4 p** | .825 | .683 | .464 | .759 | .925 | .550 | .181 | 59 |
| **n** | 59 | 59 | 59 | 59 | 45 | 59 | 59 | .181 |
| **PHQ-2 r** | .024 | -.218 | -.088 | .244 | -.146 | -.013 | -.072 | -.072 |
| **T3/T4 p** | .853 | .086 | .493 | .054 | .311 | .920 | .573 | .573 |
| **n** | 63 | 63 | 63 | 63 | 50 | 60 | 63 | 63 |
| T1, T2, T3, T4 = time point 1, 2, 3 and 4; r = Spearman-Rho (correlation-coefficient); PHQ-2 = separate module of the PHQ-4 = (Patient Health Questionnaire) assessing depression; cut off (PHQ-2) for clinically relevant depression: ≥ 3, *: p ≤ .050, **: p ≤ .010, ***: p ≤ .001 | | | | | | | | |

***Table 8S:* Correlation analyses of resource variables of interest with outcome parameter PHQ-2 at time point of interest (T1, T2, T3, T4 and longitudinal sample at T1-T4)**

| time point of interest | sufficient equipment | sufficient staff | sufficient recovery | general optimism | SOC | ESSI-D | trust in colleagues | protection by local authorities | protection by employer |
| --- | --- | --- | --- | --- | --- | --- | --- | --- | --- |
| **PHQ-2** r  **T1**  p  n | -.197***  **<.001**  605 | -.110**  **.007**  605 | -.312***  **<.001**  605 | -.312***  **<.001**  642 | -.504***  **<.001**  646 | -.282***  **<.001**  648 | -.191***  **<.001**  582 | -.220***  **<.001**  649 | -.184***  **<.001**  649 |
| **PHQ-2** r  **T2** p  n | -.090  .052  466 | -.170***  **<.001**  466 | -.464***  **<.001**  466 | -.280***  **<.001**  560 | -.550***  **<.001**  560 | -.410***  **<.001**  566 | -.300***  **<.001**  466 | -.204***  **<.001**  567 | -.188***  **<.001**  567 |
| **PHQ-2**  r  **T3** p  n | -  -  - | -.254***  **<.001**  219 | -.517***  **<.001**  219 | -.333***  **<.001**  219 | -.565***  **<.001**  219 | -.364***  **<.001**  219 | -.246***  **<.001**  219 | -.142*  **.035**  219 | -.158*  **.019**  219 |
| **PHQ-2** r  **T4** p  n | -  -  - | -.224***  **<.001**  218 | -.549***  **<.001**  218 | -.306***  **<.001**  216 | -.561***  **<.001**  216 | -.262***  **<.001**  218 | -.316***  **<.001**  218 | -.163*  **.023**  203 | -.242***  **<.001**  203 |
| **longitudinal sample** |  |  |  |  |  |  |  |  |  |
| **PHQ-2 r** | -.049 | -.091 | -.365*** | -.365*** | -.455** | -.215** | -.125 | -.096 | -.175* |
| **T1/T1 p** | .573 | .294 | <.001 | <.001 | <.001 | .007 | .152 | .234 | .029 |
| **n** | 135 | 135 | 135 | 154 | 155 | .007 | 132 | 156 | 156 |
| **PHQ-2 r** | -.138 | -.151 | -.389*** | -.299** | -.398*** | -.283** | -.206* | .018 | -.298** |
| **T1/T2 p** | .168 | .132 | **<.001** | **.001** | **<.001** | **.002** | **.042** | .849 | **.002** |
| **n** | 101 | 101 | 101 | 117 | 101 | 117 | 98 | 118 | 118 |
| **PHQ-2 r** | -.233 | -.293 | -.522*** | -.352* | -.398** | -.269 | -.222 | -.096 | -.094 |
| **T1/T3 p** | .123 | .051 | **<.001** | **.011** | **.003** | .054 | .143 | .492 | .505 |
| **n** | 45 | 45 | 45 | 51 | 52 | 52 | 45 | 53 | 53 |
| **PHQ-2 r** | -.253 | .018 | -.429* | -.307 | -.226 | -.159 | .398* | -.135 | -.162 |
| **T1/T4 p** | .203 | .929 | **.025** | .061 | .172 | .339 | **.040** | .414 | .323 |
| **n** | 27 | 27 | 27 | 38 | 38 | 38 | 27 | 39 | 39 |
| **PHQ-2 r** | -.139 | -.211 | -.452** | -.255* | -.589*** | -.417** | -.096 | -.149 | -.056 |
| **T2/T3 p** | .352 | .155 | **.001** | **.036** | **<.001** | **<.001** | .522 | .224 | .649 |
| **n** | 47 | 47 | 47 | 68 | 68 | 68 | 47 | 68 | 68 |
| **PHQ-2 r** | .080 | .095 | -.500** | -.288* | -.364** | -.329* | .178 | -.081 | -.146 |
| **T2/T4 p** | .657 | .599 | **.003** | **.027** | **.005** | **.011** | .321 | .541 | .271 |
| **n** | 33 | 33 | 33 | 59 | 59 | 59 | 33 | 59 | 59 |
| **PHQ-2 r** | - | .157 | -.300* | -.347** | -.405*** | -.212 | .047 | -.122 | -.255* |
| **T3/T4 p** | - | .219 | **.017** | **.005** | **<.001** | .096 | .712 | .339 | **.044** |
| **n** | - | 63 | 63 | 63 | 63 | 63 | 63 | 63 | 63 |
| T1, T2, T3, T4 = time point 1, 2, 3 and 4; SOC = sense of coherence; ESSI-D = ENRICHD Social Support Inventory; PHQ-2 = separate module of the PHQ-4 (Patient Health Questionnaire) assessing depression; cut off (PHQ-2) for clinically relevant depression: ≥ 3; *: p ≤ .05, **: p ≤ .01, ***: p ≤.001; “-“: not assessed at this time point | | | | | | | | | |

***Table 9S:* Parameters of external, individual and inter-relational resources of psychotherapists (PT) compared with the comparison sample (CS) at T1, T2, T3 and T4**

|  | sample | general optimism (mean) (SD) | SOC  (mean) (SD) | ESSI-D (mean) (SD) | sufficient protective gear consent (%) | sufficient staff consent (%) | sufficient recovery consent (%) | trust in colleagues consent (%) | protection by local authorities consent (%) | protection by employer consent (%) |  |
| --- | --- | --- | --- | --- | --- | --- | --- | --- | --- | --- | --- |
|  | variable of interest |  |  |  |  |  |  |  |  |  |  |
| time point |  | (n) | (n) | (n) | (n) | (n) | (n) | (n) | (n) | (n) |  |
| T1 | PT | 5.095 (1.228) | 15.633 (3.329) | 21.012 (3.748) | 56.8 | 57.5 | 42.4 | 72.4 | 53.2 | 48.4 |  |
|  |  | (642) | (646) | (648) | (634) | (633) | (634) | (609) | (667) | (667) |  |
|  | CS | 5.220 (1.346) | 15.485 (3.773) | 20.593 (4.041) | 49.1 | 55.2 | 40.3 | 70.0 | 39.1 | 45.9 |  |
|  |  | (3065) | (3069) | (3081) | (2766) | (2766) | (2766) | (2756) | (3259) | (3256) |  |
|  | statistics  T/x²,  CD/CV  p | -2.243  1.362  .025 | 1.002  3.700 .  .316 | 2.565,  3.992  .010** | 12.304  .060  <.001*** | 1.068,  .018,  .301 | .959,  .017  .328 | 1.445,  .021,  .229 | 45.547  .108  <.001*** | 1.473,  .019  .225 |  |
| T2 | PT | 5.150 (1.267) | 15.384 (3.581) | 20.917 (3.862) | 79.0 | 40.5 | 33.1 | 70.2 | 35.8 | 49.4 |  |
|  |  | (560) | (560) | (566) | (486) | (486) | (486) | (486) | (581) | (581) |  |
|  | CS | 5.130 (1.367) | 14.901 (3.724) | 20.903 (3.966) | 73.7 | 26.5 | 26.6 | 67.4 | 26.3 | 41.7 |  |
|  |  | (1352) | (1359) | (1367) | (1164) | (1164) | (1164) | (1164) | (1457) | (1455) |  |
|  | statistics  T/x²  CD/CV  p | .236,  1.339  .813 | 2.613,  3.683  .009** | .072,  3.935  .942 | 5.176  .056  .023* | 31.553  .138  <.001*** | 7.091  .066  .008** | 1.248  .028,  .264 | 18.258  095  <.001*** | 9.942  .070  <.001*** |  |
| T3 | PT | 5.023 (1.202) | 15.566 (3.464) | 21.055 (3.578) | **** | 29.2 | 32.9 | 73.5 | 49.3 | 63.5 |  |
|  |  | (219) | (219) | (219) |  | (219) | (219) | (219) | (219) | (219) |  |
|  | CS | 5.140 (1.342) | 15.307 (3.787) | 20.745 (4.108) | **** | 23.3 | 29.9 | 67.4 | 35.8 | 55.7 |  |
|  |  | (1638) | (1638) | (1638) |  | (1638) | (1637) | (1638) | (1638) | (1638) |  |
|  | statistics  T/x²,  CD/CV  p | -1.347  1.326  .179 | -4.002  3.750  .338 | -2.007  4.049  .239 | **** | 3.688  .045  .055 | .827  021  .363 | 3.328  .042  .068 | 15.130  .090  <.001*** | 4.702  .050  .030 |  |
| T4 | PT T4 | 4.931 (1.350) | 15.426 (3.237) | 21.101 (3.642) | **** | 15.6 | 31.6 | 71.1 | 30.9 | 53.1 |  |
|  |  | (216) | (216) | (218) |  | (225) | (225) | (225) | (207) | (207) |  |
|  | CS T4 | 5.000 (1.439) | 14.867 (3.807) | 20.758 (4.267) | **** | 13.0 | 27.5 | 63.3 | 18.5 | 47.1 |  |
|  |  | (1668) | (1670) | (1680) |  | (1848) | (1848) | (1848) | (1450) | (1450) |  |
|  | statistics  T/X²  CD/CV  p | -.701  1.429  .483 | 2.339  3.746  .020* | 1.280  4.200  .202 | **** | 1.150  024  .284 | 1.648  .028  .199 | 5.377  . 051  .020* | 17.483  .103  <.001*** | 2.645  .040  104 |  |
| statistics CS ANOVA (factor: time point)  F  η_p_^2^  p | | 8.774  .003  <.001*** | 13.455  .005  <.001*** | 1.970  .000  .116 | χ^2^-test  X², CV, p | | | | | | |
| T1/T2 p Games-Howell post hoc test | | .252 | <.001*** | .079 | X² 202.414  CV .227  p <.001*** | X² 270.992  CV .263  p <.001*** | X² 66.332  CV .130  p <.001*** | X² .600  CV .026  p .107 | X² 72.438  CV .124  p <.001*** | X² 6.962  CV .038  p .008 |  |
| T1/T3 p Games-Howell post hoc test | | .255 | .417 | .612 | **** | X² 426.815  CV .311  p <.001*** | X² 48.392  CV .105  p <.001*** | X² .141  CV .027  p .076 | X² 5.090  CV .032  p .024* | X² 42.605  C. .093  p <.001 |  |
| T1/T4 p Games-Howell post hoc test | | <.001*** | <.001*** | .560 | **** | X² 836.903  CV .426  p <.001*** | X² 79.871  CV .132  p <.001*** | X² 22.546  CV .070  p <.001*** | X² 193.536  CV .203  p <.001*** | X² .630  CV .012  P .427 |  |
| T2/T3 p Games-Howell post hoc test | | .999 | .017* | .711 | **** | X² 3.810  CV .037  p .051 | X² 3.501  CV .035  p .061 | X² .001  CV .000  p .980 | X² 32.280  CV .102  p <.001*** | X² 60.605  CV .140  p <.001*** |  |
| T2/T4 p Games-Howell post hoc test | | .050* | .995 | .769 | **** | X² 88.095  CV .171  p <.001*** | X² .265  CV .009  p .607 | X² 5.257  CV .042  p .022 | X² 25.468  CV .094  p <.001*** | X² 8.531  CV .054  p .003** |  |
| T3/T4 p Games-Howell post hoc test | | .023* | .005** | 1.000 | **** | X² 63.260  CV .135  p <.001*** | X² 2.413  CV .026  p .120 | X² 6.565  CV .043  p .010** | X² 114.958  CV .193  p <.001*** | X² 22.967  CV .086  p <.001*** |  |
| PT = psychotherapists; CS = comparison sample; SOC = sense of coherence; ESSI-D = ENRICHD Social Support Inventory; significant p-values are marked: *: p ≤ .050, **: p ≤.010, ***: p ≤ .001*; Bonferroni correction: p ≤.050/4 (.013) for four score variables with means (PHQ-2-score, general optimism, SOC, ESSI-D) and p ≤ .050/7.007 for seven categorial variables ( variablePHQ-2 rate + six resource-variables), η_p_^2^ = partial Eta²; CV = Cramer`s V; X² = Chi Square; PHQ-2 = separate module of the PHQ-4 (Patient Health Questionnaire) assessing depression; cut off (PHQ-2) for clinically relevant depression: ≥ 3; T1, T2, T3, T4 = time point 1, 2, 3 and 4; ****: not assessed at this time point | | | | | | | | | | | |
